# Marine Atmospheric Corrosion of Carbon Steel: A Review

**DOI:** 10.3390/ma10040406

**Published:** 2017-04-13

**Authors:** Jenifer Alcántara, Daniel de la Fuente, Belén Chico, Joaquín Simancas, Iván Díaz, Manuel Morcillo

**Affiliations:** National Centre for Metallurgical Research (CENIM/CSIC), Avda. Gregorio del Amo n° 8, 28040 Madrid, Spain; j.alcantara@cenim.csic.es (J.A.); delafuente@cenim.csic.es (D.d.l.F.); bchico@cenim.csic.es (B.C.); jsimancas@cenim.csic.es (J.S.); ivan.diaz@cenim.csic.es (I.D.)

**Keywords:** atmospheric corrosion, marine environment, carbon steel

## Abstract

The atmospheric corrosion of carbon steel is an extensive topic that has been studied over the years by many researchers. However, until relatively recently, surprisingly little attention has been paid to the action of marine chlorides. Corrosion in coastal regions is a particularly relevant issue due the latter’s great importance to human society. About half of the world’s population lives in coastal regions and the industrialisation of developing countries tends to concentrate production plants close to the sea. Until the start of the 21st century, research on the basic mechanisms of rust formation in Cl^−^-rich atmospheres was limited to just a small number of studies. However, in recent years, scientific understanding of marine atmospheric corrosion has advanced greatly, and in the authors’ opinion a sufficient body of knowledge has been built up in published scientific papers to warrant an up-to-date review of the current state-of-the-art and to assess what issues still need to be addressed. That is the purpose of the present review. After a preliminary section devoted to basic concepts on atmospheric corrosion, the marine atmosphere, and experimentation on marine atmospheric corrosion, the paper addresses key aspects such as the most significant corrosion products, the characteristics of the rust layers formed, and the mechanisms of steel corrosion in marine atmospheres. Special attention is then paid to important matters such as coastal-industrial atmospheres and long-term behaviour of carbon steel exposed to marine atmospheres. The work ends with a section dedicated to issues pending, noting a series of questions in relation with which greater research efforts would seem to be necessary.

## 1. Introduction

Steel is the most commonly employed metallic material in open-air structures, being used to make a wide range of equipment and metallic structures due to its low cost and good mechanical strength. Much of the steel that is manufactured is exposed to outdoor conditions, often in highly polluted atmospheres where corrosion is much more severe than in clean rural environments.

The atmospheric corrosion (AC) of carbon steel (CS) is an extensive topic that has been studied by many researchers. Useful books and chapters have been published by a number of authors [1,2,3,4,5,6,7,8].

Since the 1920’s much time and effort has been devoted to studying the corrosion of metals in natural atmospheres. As a result, the importance of various meteorological and pollution parameters on metallic corrosion in now fairly well known. The effect of sulfur dioxide (SO_2_) on AC has been widely studied, but until relatively recently researchers have paid surprisingly little attention to the action of marine chlorides in AC, despite it being well known that airborne salt in coastal regions promotes a marked increase in AC rates compared to clean atmospheres.

The issue of corrosion in coastal regions is particularly relevant in view of the latter’s great importance to human society. About half of the world’s population lives in coastal regions and the industrialisation of developing countries tends to concentrate production plants close to the sea.

The first rigorous study on the salinity of marine atmospheres and its effect on metallic corrosion was carried out in Nigeria by Ambler and Bain [9] and dates from 1955. For many years it was simply accepted that marine chlorides dissolved in the aqueous adlayer considerably raised the conductivity of the electrolyte on the metal surface and tended to destroy any passivating films. In 1973 Barton noted that the mechanism governing the effects of chloride ions (Cl^−^) in AC had not been completely explained, and that the higher corrosion rate of steel in marine atmospheres could also be due to other causes, such as: (a) the hygroscopic nature of Cl^−^ species (sodium chloride (NaCl), calcium chloride (CaCl_2_), magnesium chloride (MgCl_2_)), which promotes the electrochemical corrosion process by favouring the formation of electrolytes at relatively low relative humidity (RH); and (b) the solubility of the corrosion products. Thus, in the case of iron, which does not form stable basic chlorides, the action of chlorides is more pronounced than with other metals (zinc, copper, etc.) whose basic salts are only slightly soluble [4].

In the year 2000, Nishimura et al. noted that with the exception of a few studies, research on the basic mechanisms of rust formation in Cl^−^-rich marine atmospheres had been rather scarce [10]. Since then, scientific knowledge of marine atmospheric corrosion (MAC) has advanced greatly, perhaps as a result of the need to develop new weathering steels (WS) with greater MAC resistance than conventional WS, whose main limitation is precisely their low corrosion resistance in this type of environment [11]. This hypothesis seems to be confirmed by the high proportion of MAC studies that consider this type of materials.

Therefore, this is a relatively young scientific field and there continue to be great gaps in its comprehension [12]. Nevertheless, in the authors’ opinion a considerable body of knowledge has been built up in a large number of published scientific papers, and it is now time to make an up-to-date review of the current state-of-the-art and to assess what issues still need to be addressed. That is the purpose of the present review.

## 2. Basic Concepts

The AC of metals is an electrochemical process which is the sum of individual processes that take place when an aqueous adlayer forms on the metal. This electrolyte can be either an extremely thin moisture layer (just a few monolayers) or an aqueous film of hundreds of microns in thickness (when the metal is perceptibly wet). Aqueous precipitation (rain, fog, etc.) and humidity condensation due to temperature changes (dew), capillary condensation when the surfaces are covered with corrosion products or with deposits of solid particles, and chemical condensation due to the hygroscopic properties of certain polluting substances deposited on the metallic surface, are the main promoters of metallic corrosion in the atmosphere [2]. Recent studies on the wetting of metal surfaces in order to understand the process controlling AC, as well as the effect of RH on steel corrosion in the presence of sea salt aerosols (NaCl and MgCl_2_) can be found in references [13,14,15,16].

The magnitude of AC is basically controlled by the length of time that the surface is wet, though it ultimately depends on a series of factors such as RH, temperature, exposure conditions, atmospheric pollution, metal composition, rust properties, etc. [5,17]. The AC process involves simultaneous oxidation and reduction reactions which can be accompanied by other chemical reactions in which the corrosion products may take part.

The anodic reaction, consisting of the oxidation of the metal, can be given as:

Fe→Fe^2+^ + 2e^−^, (1)

Oxygen (O_2_), which is highly soluble in the aqueous layer, is a possible electron acceptor. Oxygen reduction in neutral or basic media takes place according to the reaction:

O_2_ + 2H_2_O + 4e^−^→4OH^−^, (2)

The hydroxide ions migrate to anodic areas, forming ferrous hydroxide [Fe(OH)_2_] as the initial corrosion product.

Oxygen diffusion through the aqueous adlayer is usually a corrosion rate-controlling factor. The corrosion rate reaches a maximum value for intermediate thicknesses of the aqueous adlayer on the metal surface. The joining up of individual droplets to form relatively thick electrolyte layers somewhat reduces the rate of attack, as it hampers the arrival of oxygen. On the other hand, an excessive decrease in the moisture layer thickness halts the corrosion process, due to the high ohmic resistance of very thin layers where the ionisation and dissolution reactions of the metal are obstructed. Fast drying and repeated wetting of the surface leads to stronger corrosion effects. During drying periods, the convective currents caused by evaporation of the electrolyte lead to a decrease in the effective thickness of the diffusion layer, with the consequent rise in the transportation rate of cathodic depolariser, thus making the corrosion rate a cathodically controlled process. The electrolyte is self-stirring during evaporation [3,18].

Another factor that substantially determines the intensity of the corrosive phenomenon is the chemical composition of the atmosphere (air pollution by gases, acid vapours or seawater aerosols). SO_2_ and NaCl are the most common corrosive agents in the atmosphere. Nitrogen oxides (NO_*x*_) are another important source of atmospheric pollution.

### 2.1. Sulfur Dioxide

The effect of SO_2_ on AC has been studied by many authors [7]. SO_2_ is often found in the atmosphere in concentrations that vary considerably depending on the type of industries in the region, the presence of power plants, time of year, etc. SO_2_ is much more aggressive to steel when its concentration exceeds 0.1 mg·m^−3^, a level that is easily reached in many towns, especially in winter. Fortunately, the SO_2_ concentration in urban air has decreased greatly in recent years due to efforts to reduce pollution [19].

Rozenfeld [3] has shown that SO_2_ is also an active cathodic depolarising agent due to its susceptibility to be reduced on metals. SO_2_ is some 2600 times more soluble in water than oxygen, so even if the SO_2_ gas content in the atmosphere is very small, its concentration in the electrolyte and its effect can be similar to that of oxygen, which is the depolarising agent par excellence. Thus, above a certain acidity level in polluted atmospheres, SO_2_ can act as an oxidising agent and greatly accelerate the cathodic process.

Rainwater can absorb SO_2_ from the atmosphere as it falls, giving rise to what is known as acid rain. For this reason the pH of rainwater collected downwind of highly industrialised regions of Europe sometimes presents clearly acid values, as in Norway [20], where average daily and monthly measurements of down to pH 2.9 have been recorded. In such situations, the cathodic reaction of hydrogen evolution can be relevant.

2H^+^ + 2e^−^→H_2_, (3)

Kucera [21] distinguishes between the rinsing effect of rainwater, which tends to wash away pollutants that accumulate on the metallic surface, and the harmful effect of acid precipitation. In terms of corrosion, Kucera suggests a predominance of the rinsing effect in appreciably polluted areas, whereas in rural areas rainwater with a circumstantially low pH may worsen the situation.

**Figure 1:**
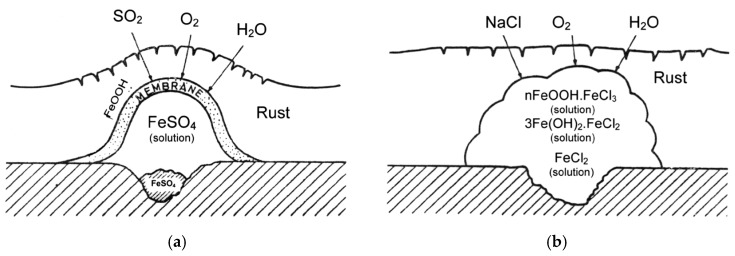
Schematic representation of a sulphate nest (**a**) and a chloride agglomeration (**b**) [22].

SO_2_ gives rise to the formation and propagation of sulfate “nests”, according to reactions (4,5), which start to appear at isolated points on the surface but whose number increases until all the surface is coated with a rust film (Figure 1a) [22].

SO_2_ + H_2_O + ½O_2_→H_2_SO_4_, (4)

2H_2_SO_4_ + 2Fe + O_2_→2H_2_O + 2FeSO_4_, (5)

Hydrolysis of the ferrous sulfate formed in these nests controls their propagation (reactions 6,7).

6FeSO_4_ + H_2_O + 3/2O_2_→2Fe_2_(SO_4_)_3_ + 2FeOOH, (6)

Fe_2_(SO_4_)_3_ + 4H_2_O→2FeOOH + 3H_2_SO_4_, (7)

Osmotic pressure may cause the nests to burst, thus raising the corrosion rate [22].

### 2.2. Saltwater Aerosols

The deposition of salt particles on a metallic surface accelerates its corrosion, especially, as in the case of chlorides, if they can give rise to soluble corrosion products rather than the only slightly soluble products formed in pure water.

Cl^−^ ions are abundant in marine atmospheres, where the fundamental source of mineralisation consists of saltwater particles that are carried along by air masses as they pass over seas, oceans and salt lakes [3]. According to Ambler and Bain [9], only salt particles and droplets of more than 10 µm cause corrosion when deposited on a metallic surface. Given that such particles remain in the atmosphere for a short time, usually corrosion completely loses its marine character just a few kilometres inland.

For salt to accelerate corrosion the metallic surface needs to be wet. The RH level that marks the point at which salt starts to absorb water from the atmosphere (hygroscopicity) seems to be critical from the point of view of corrosion.

As has been noted above, the effect of Cl^−^ ions on CS corrosion mechanisms has been much less widely studied than the effect of SO_2_. A high Cl^−^ concentration in the aqueous adlayer on the metal and high moisture retention in very deteriorated areas of the rust give rise to the formation of ferrous chloride (FeCl_2_), which hydrolyses the water:

FeCl_2_ + H_2_O→FeO + 2HCl, (8)

Notably raising the acidity of the electrolyte. In this situation the cathodic reaction (3) becomes important, accelerating the corrosion process. The anolyte on the steel surface and in the pits that have formed becomes saturated (or close to saturation) with the highly acidic FeCl_2_ solution. Both the metallic cations and hydrogen ions require neutralisation, which occurs by the entry of Cl^−^ ions, but this leads to an increase in the Cl^−^ concentration which intensifies metal dissolution, giving rise in turn to the entry of more Cl^−^, which further intensifies the corrosion process. This attack mechanism is fed by the corrosion products themselves (feedback mechanism), and it is sometimes referred to as “autocatalytic” [23].

Unlike SO_2_ pollution, Cl^−^ pollution does not cause the formation of nests but Cl^−^ agglomerates. The literature also sometimes mentions the formation of “chloride nests” [24], but the osmotic pressure of FeCl_2_ or NaCl does not influence corrosive activity, which instead is determined by other causes such as the ability of ferrous and ferric chlorides to form complexes (nFeOOH·FeCl_3_) or a solution of FeCl_3_ in FeOOH in the form of a gel. No amorphous oxide/hydroxide membrane is originated (Figure 1b) [22].

### 2.3. Hydrogen Chloride Vapours

Askey et al. [25] published an interesting study of iron corrosion by atmospheric hydrogen chloride (HCl), in which they suggest a direct reaction between CS and HCl. HCl reacts directly with the metal to produce soluble FeCl_2_, which is then oxidised to FeOOH, releasing HCl.

Fe + 2HCl + ½O_2_⇌FeCl_2_ + H_2_O, (9)

2FeCl_2_ + 3H_2_O + ½O_2_⇌2FeOOH + 4HCl, (10)

It should be noted that this is a reaction cycle in which rereleased HCl reacts with iron to form fresh FeCl_2_. Once started, therefore, the cycle is independent of incoming HCl. Corrosion continues until the Cl^−^ ions are removed (possibly by washing away of FeCl_2_) after which fresh incoming HCl reacts with the metal surface and reinitiates the cycle. The cycle proposed above is analogous to the acid regeneration cycle proposed by Schikorr [26] for the action of SO_2_ on iron.

## 3. Experimentation on Marine Atmospheric Corrosion

Most studies of MAC have involved the performance of field exposure tests. Specimens of appropriate dimensions are mounted on racks using porcelain or plastic insulating clips. The exposure angle is generally 45° to the horizontal in Europe or 30° to the horizontal in the United States. It is general practice to have the panel racks facing south in the northern hemisphere or north in the southern hemisphere. However, when panels are mounted in coastal locations it is desirable to have the racks facing the shore. Further to these general requirements it is advised to follow the appropriate specific standards that have been published [27,28].

Atmospheric exposure tests usually involve the use of flat specimens of metals and alloys, although wire specimens are sometimes also used, such as in the ISOCORRAG International Atmospheric Exposure Program [29]. It is increasingly common to use both flat and wire specimens to evaluate the aggressivity (corrosivity) of atmospheres [30]. It is also interesting to note the quick response and high sensitivity of the wire-on-bolt technique, using specimens originally devised by Bell Telephone Laboratories [31] and subsequently developed by the Canadian company Alcan International [32]. This technique consists of the atmospheric exposure for just three months of metallic wires wound firmly around bolts of another metal. The functioning of the galvanic couple depends among other factors on the atmosphere where it is exposed. Doyle and Godard report that aluminium wire wound around an iron bolt is highly sensitive to the marine atmosphere [33].

The specimens are exposed to the atmosphere for a given time and subsequently analysed in the laboratory by gravimetric techniques to determine the corrosion losses experienced. Mass-loss data allows structural integrity to be estimated after a given number of years of service. Structural engineers typically use mass-loss data to overbuild a structure, allowing for a given mass loss over the predicted lifetime.

Over the course of time different accelerated tests have been developed to simulate AC in the laboratory. The disadvantage of accelerated corrosion tests is that the results obtained do not always coincide with those found in real atmospheric exposure tests. For instance, the laboratory simulation of marine atmosphere exposure has long been carried out by constant exposure to salt fog. However, the classic salt fog test has a bad reputation and is unanimously considered to offer poor reproducibility and correlation with atmospheric exposure. The application of intermittent salt spray, on the other hand, is a much better approximation to marine and coastal conditions [34], and the cyclic salt fog test, along with the use of alternative saline solutions to NaCl, provides much better correlations [35].

As has been noted above, the existence and duration of wetting and drying stages play an important role in AC mechanisms. The need for wet/dry cycles to simulate AC is now well established and any accelerated laboratory test for this purpose needs to take this aspect into account. Today’s standard accelerated tests for the simulation of atmospheric exposure are all based on wet/dry cycles. Although some standards set out conditions for the performance of such tests [36], they tend to be carried out in many different ways, and the results of one researcher are not always comparable to those of another. Despite this difficulty, analysis of the data obtained by researchers throughout the world in the most varied of experimental conditions has led to great advances in the knowledge of MAC. As will be mentioned below, it would nevertheless be desirable to standardise a universal wet/dry cyclic test that could be followed by all researchers so that the results obtained would be comparable.

Considerable efforts have been made to obtain reliable estimates of AC without the limitations of gravimetric tests, especially in terms of their enormous duration. Very good results have been obtained with electrochemical cells [37]. Electrochemical techniques, and in particular impedance measurements, have been widely used by many researchers in numerous studies related with AC.

**Figure 2:**
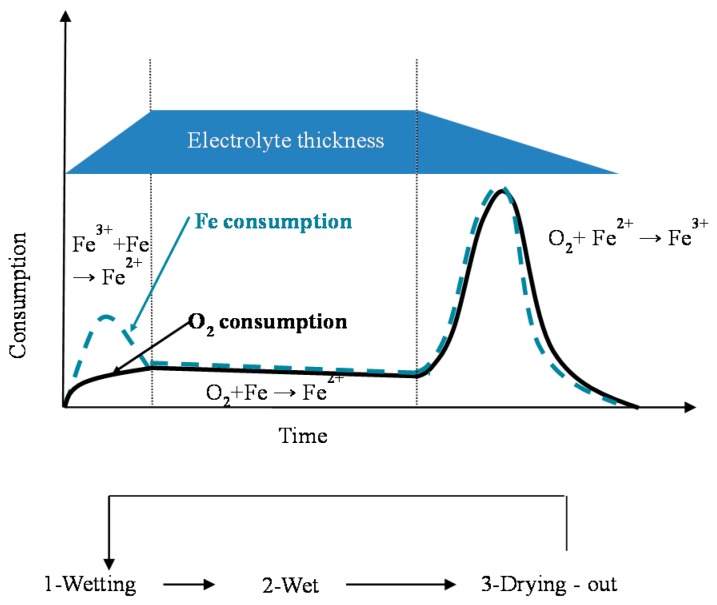
Schematic representation according to Stratmann [38] of atmospheric rusting cyclic mechanism.

In particular, attention is drawn to the important atmospheric rusting cycle mechanism proposed by Stratmann [38]. In an electrochemical study of phase transitions in rust layers, Stratmann showed that when a pre-rusted iron sample was wetted, iron dissolution was not immediately balanced by a reaction with oxygen, but rather by the reduction of the preexisting rust (lepidocrocite) with later reoxidation of the reduced species. Thus, Stratmann [38] proposed dividing the AC mechanism of pure iron into the following three stages (Figure 2): (a) wetting of the dry surface; (b) wet surface; and (c) drying-out of the surface.

Nishimura et al. [10,39] carried out a laboratory study of the electrochemical behaviour of rust formed on CS in wet/dry cycles in solutions containing Cl^−^ ions, simulating exposure in marine atmospheres. They observed that akaganeite formation was the cause which enormously accelerated the AC process in this type of atmosphere, being electrochemically reduced and consumed during the wetting of the metallic surface, in contrast to the important role played by lepidocrocite in steel corrosion in Cl^−^-free atmospheres [38].

Over the last decade, great advances have been made in the understanding of AC mechanisms. Many of these advances have been due to the research groups of Legrand [40] and Dillmann [41]. Basic research carried out in this field has been related with a greater knowledge of the electrochemical reactivity of the ferric phases that constitute atmospheric rust and the coupling or decoupling of anodic and cathodic reactions.

A fine characterisation of corrosion product layers identifying the oxide phases on a metal surface yields valuable information on the evolution of the corrosion process in a given atmosphere. Not only is it important to identify the different oxides but also to ascertain the fraction of each corrosion product and its distribution in the rust layer in order to gain a better understanding of the corrosion process. The distribution of the phases can drastically influence local corrosion mechanisms. Elemental composition can be determined by energy dispersive X-ray (EDX) analysis and electron probe microanalysis (EPMA), and several macroscopic techniques such as X-ray diffraction (XRD), infrared spectroscopy (IRS) and Mössbauer spectroscopy (MS) are commonly used for corrosion products characterisation. However, it may be very important to obtain a fine and local determination of the structure of corrosion products in order to understand corrosion mechanisms. In such cases the local structure of the corrosion layers must be characterised with the help of microprobes: Raman microspectroscopy (µRS), X-ray microdiffraction (µXRD), X-ray absorption spectroscopy (XAS), etc. The specificities of each analysis method strongly influence the type of phase identified [11].

XRD is one of the most commonly used techniques for identifying the rust composition and the structure of different components in corrosion products. One of the limitations of XRD is the separate identification of magnetite and maghemite. Both oxides have a cubic structure and nearly identical lattice parameters at room temperature, making them nearly indistinguishable by XRD. However, their magnetic and electric properties are quite different, thereby allowing MS to identify each. According to Cook [42], corrosion research is one area in which MS has become a required analytical technique. This is in part due to the need to identify and quantify the nanophase iron oxides that are nearly transparent to most other spectroscopic techniques.

Rust composition studies using µRS usually demand a high laser power for the excitation of spectra because some of the most common iron oxides and oxyhydroxides are poor light scatterers. Sample degradation frequently occurs under intense sample illumination and may lead to the misinterpretation of spectra. Low laser power minimises the risks of spectral changes due to sample degradation [43]. Moreover, in some cases, particularly when the phases are less crystallised, it is difficult to discriminate one phase from another only by µRS because the Raman shift is very close.

According to Monnier et al., the use of complementary analytical techniques (µXRD, XAS and MS) is needed to obtain accurate Raman phase characterisation. Each technique provides complementary information. µXRD is more sensitive to crystallised phases while µRS presents a higher spatial resolution and allows the detection and location of crystallised phases (goethite, lepidocrocite, maghemite, akaganeite) from less crystallised ones (feroxyhyte, ferrihydrite). Discrimination of maghemite, feroxyhyte and ferrihydrite could be partially solved by the use of XAS [44].

Knowledge of the rust layer structure is another aspect widely studied by researchers. The techniques traditionally used are optical microscopy (OM), polarised light microscopy, scanning electron microscopy (SEM) and transmission electron microscopy (TEM)/electron diffraction (ED). In order to characterise corrosion product structures in various scales, Kimura et al. [45] use several analytical approaches that are sensitive to three structural-correlation lengths: long-range order (LRO) (>50 nm), middle-range order (MRO) (~1–50 nm), and short-range order (SRO) (<1 nm) and Konishi et al. employ X-ray absorption fine structure analysis (XAFS) methods, including extended XAFS and X-ray absorption near-edge structure (XANES), for characterisation of rust layers formed on Fe, Fe-Ni and Fe-Cr alloys exposed to Cl^−^-rich environments [46].

Complementary analyses on the porosity of rust layers have also been conducted by several researchers. Dillmann et al. use various techniques such as small-angle X-ray scattering (SAXS), Brunauer-Emmett-Teller (BET) and mercury intrusion porosimetry (MIP) [47]. Attention is also drawn to the studies of Ishikawa et al., where the specific surface area of the pores was calculated by fitting the BET equation to N_2_ adsorption isotherms [48].

Over the past few decades, the new analytical techniques developed to study properties of solid surfaces, such as chemical composition, oxidation state, morphology, structure, etc. have continued to increase and improve in terms of resolution and sensitivity. The more recent analytical techniques are both surface-sensitive and able to provide information under in-situ conditions. According to Leygraf et al. [8], it is anticipated that the number and variety of in-situ techniques for probing surfaces will continue to increase.

## 4. The Marine Atmosphere

From the point of view of MAC, the marine atmosphere is characterised by the presence of marine aerosol. Cl^−^ ions are abundant in marine atmospheres, where the fundamental source of mineralisation consists of saltwater particles that are carried along by air masses as they pass over seas, oceans, and salt lakes. Marine salts are mainly NaCl, but quite appreciable amounts of potassium, magnesium and calcium ions are also found in rainfall.

### 4.1. Atmospheric Salinity

Atmospheric salinity is a parameter related with the amount of marine aerosol present in the atmosphere at a certain geographic point. Marine aerosols, consisting of wet aerosols, partially wet aerosols and non-equilibrium aerosols depending on the atmospheric humidity, are carried along by the wind and can come into contact with metallic structures and greatly accelerate the corrosion process. The sizes of the three types of aerosols and the resultant dry aerosols were estimated by Cole et al. [49].

Salinity in marine atmospheres varies within very broad limits (<5 to >300 mg Cl^−^/m^2^·d) [50]. While extremely high values have been recorded close to surf, salinity at other points on the shoreline near calmer waters is no more than moderate. The concentration of marine aerosol decreases with altitude [51,52]. Meira et al. find that this relationship can be represented by an exponential decrease function which is influenced by the wind regime [52].

An increase in wind speed, even on the same coast, does not always lead to an increase in salinity, as the final result is dependent on the wind direction. In fact, an increase in wind speed can even reduce the degree of pollution by purifying the exposure site of pollutant. This will naturally depend on the situation of the exposure site in relation with the sea, and on the direction and type of winds blowing at a given time.

Marine aerosol is comprised of fine particles suspended in the air (jet drops, film drops, brine drops and sea-salt particles), solid or liquid, whose sizes vary from a few angstroms to several hundred microns in diameter [53]. Marine aerosol particles are usually classified by size into two classes: coarse particles, with an equivalent aerodynamic diameter of >2 µm; and small particles, with a diameter of <2 µm. Fine particles are in turn subdivided into Aitken nuclei (<0.05 µm) and particles formed by accumulation (with diameters of between 0.05 and 2 µm). In coastal locations (<2 km from the seashore) the most common aerosols deposited are in the coarse size range: 2–100 µm in diameter [54,55].

Large marine aerosol particles (diameter >10 µm) remain for only a short time in the atmosphere; the larger the particle size, the shorter the time. On the other hand, particles of a diameter of <10 µm may travel hundreds of kilometres in the air without sedimenting [9,56].

Li and Hihara [55] in a study of natural salt particle deposition on CS for only 30 min in a severe marine site in Hawaii, found that most airborne salt particles had diameters ranging from approximately 2–10 µm, and varied in composition ranging from almost pure NaCl or KCl to mixtures of NaCl, KCl, CaCl_2_ and MgCl_2_. These differences in composition may depend on whether the seawater droplets dehydrate, crystallise and fragment while airborne, or if they are deposited as liquid droplets before crystallising.

### 4.2. Production of Marine Aerosol

Cole et al. described marine aerosol formation, chemistry, reaction with atmospheric gases, transport, deposition onto surfaces, and reaction with surface oxides [57].

The wind, which stirs up and carries along seawater particles, is the force responsible for the salinity present in marine atmospheres. Oceanic air is rich in marine aerosols resulting from the evaporation of drops of seawater, mechanically transported by the wind. The origin, concentration and vertical distribution of marine aerosol over the surface of the sea has been studied by Blanchard and Woodcock [51].

The first step in the production of aerosol particles is the breaking of waves [51,58,59]. The turbulence that accompanies this phenomenon introduces air bubbles into the water which subsequently burst and launch sea salt particles into the atmosphere. On the high seas the breaking of waves depends on the speed of the wind blowing over them. In the coastal surf zone, waves can break without the need for simultaneous wind action, and the amount of aerosol generated is largely dependent on the type of sea floor (uniformity, slope, etc.) and the width of the surf zone.

Aerosol levels at the seashore depend both on the aerosol that is generated out at sea, which is carried to the coast by marine winds, and that which is generated in the surf zone close to the shoreline [60,61,62]. Of the two, the latter seems to be the main contributor to the Cl^−^ levels measured in the lower layers of the atmosphere in coastal areas [61,63].

**Figure 3:**
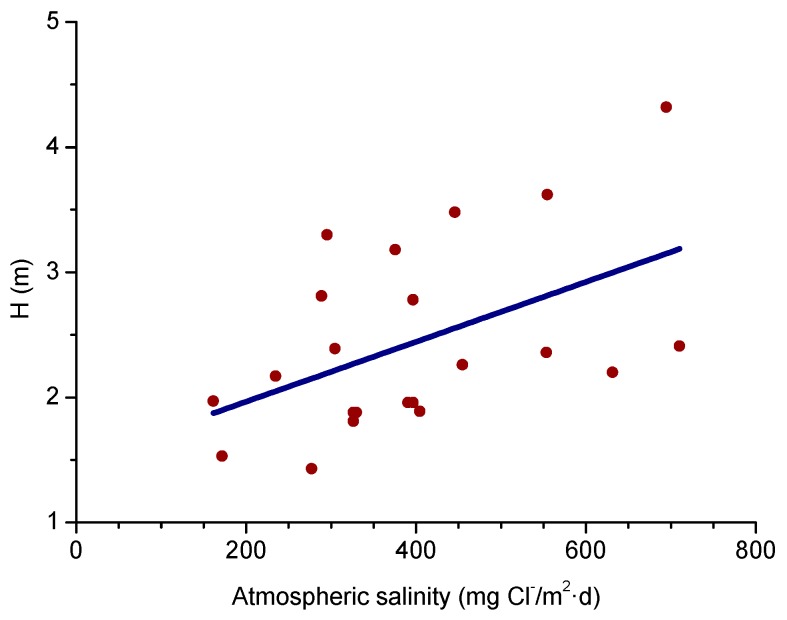
Variation of monthly average salinity with monthly average spectral wave height values (H). The regression line shows the general trend [64].

A relationship has been seen between salinity and wave height [64]. The graph in Figure 3 shows the variation of atmospheric salinity with the average spectral wave height. As can be seen in the figure, monthly average spectral wave height values of 1.5–2.0 m are sufficient to produce high monthly average salinity values of 100–200 mg Cl^−^/m^2^·d.

**Figure 4:**
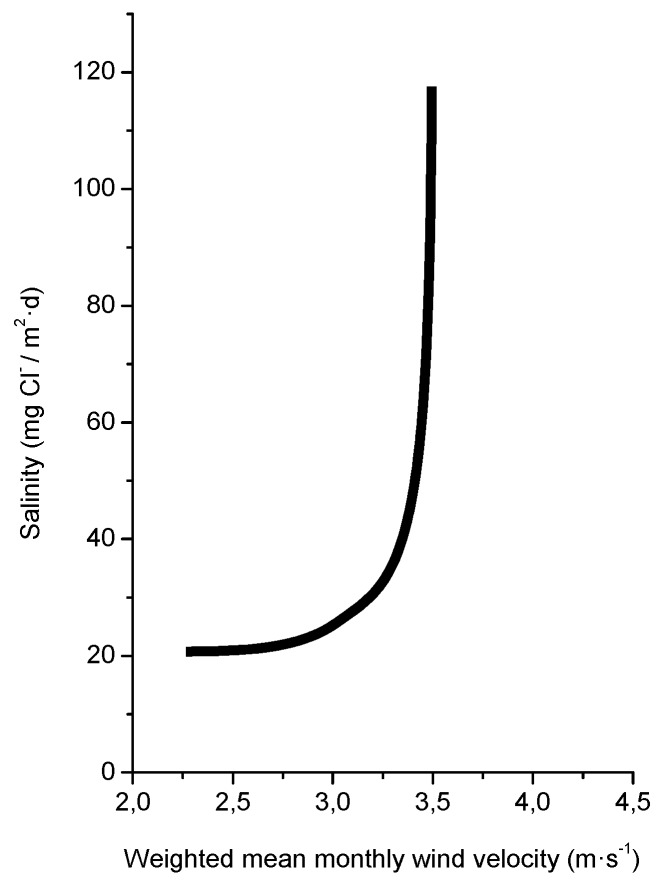
Relation between atmosphere salinity and weighted mean monthly wind velocity for marine winds [66].

Several studies in the literature have attempted to relate aerosol levels measured at high sea and on the coast with wind speed. Potential and exponential type functions express the considerable effect of this variable on marine aerosol production (especially when the wind speed exceeds some 3–5 m/s) [51,65,66,67]. In Figure 4, Morcillo et al. note that the wind only needs to blow short time at speeds above 3 m/s in directions with high entrainment of marine aerosol (they call them “saline winds”) for atmospheric salinity to reach important values [66].

### 4.3. Entrainment of Marine Aerosol Inland

Aerosol particles can be entrained inland by marine winds (winds proceeding from the sea), settling after a certain time and after travelling a certain distance. The wind regime directly influences aerosol production and transportation, and is significantly affected by geostrophic winds, large-scale atmospheric stability, and the difference between diurnal land and sea temperatures, which varies according to the season of the year. It is also dependent on the latitude, ruggedness of the coastline, and undulation of the land surface [51,58]. A reduction in the size and mass of aerosol particles due to drying of the droplets can considerably increase the entrainment distance.

**Figure 5:**
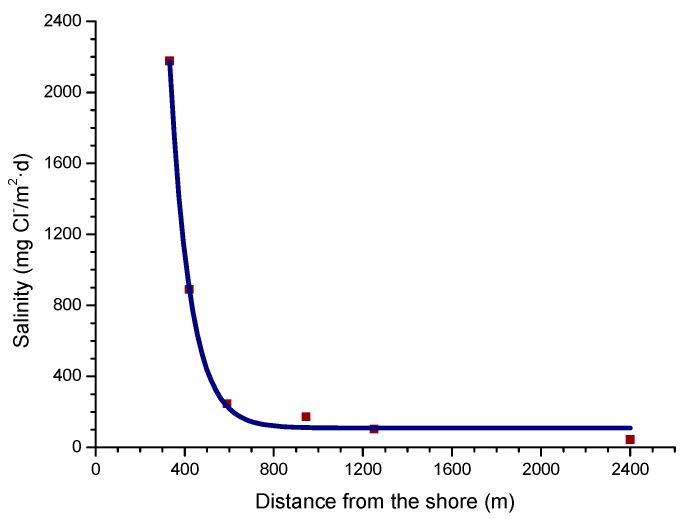
Variation in atmospheric salinity with distance from the shore [64].

In a recent study by Alcántara et al. [64], it was seen that the variation in salinity with the distance from the shoreline (Figure 5) clearly showed an exponential relationship.

Y = 78288.23 exp (−X/91.34) + 108.40,(11)

Being Y the atmospheric salinity expressed as mg Cl^−^/m^2^·d and X the distance from the shore in meters (m).

**Figure 6:**
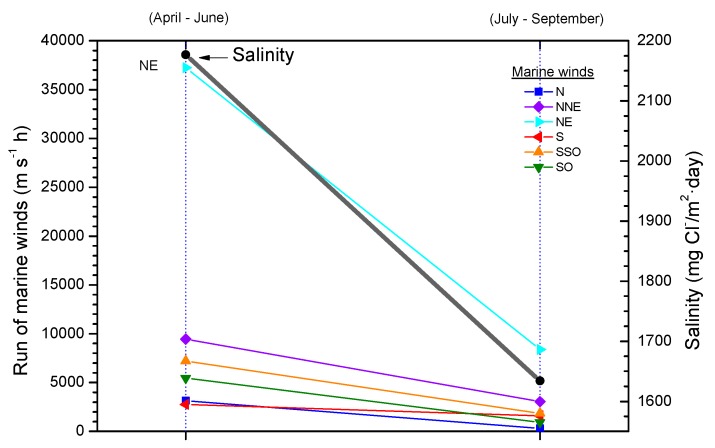
Salinity data and run of different marine winds in two three-month periods [64].

Comparison of the atmospheric salinity at an exposure site and the average wind speed often fails to yield a clear relationship between both parameters. Calculation of the average wind speed takes into account the speeds recorded in all wind directions, and not only marine winds, which it would be reasonable to suppose are those that govern the presence of marine aerosol masses in coastal regions. In this sense it would be interesting to know whether the atmospheric salinity at the site is related with the run of marine winds, which is the sum of adding together the marine wind speed in each direction multiplied by the time it has been blowing [64]. Figure 6 has been prepared accordingly and clearly explains the decrease in the salinity value obtained in the second three-month period by a decline in the run of all marine winds, especially the most frequent (north-easterly, NE). Therefore, more than the average wind speed in the study area, the total run of marine winds is the parameter that has the greatest influence on the atmospheric salinity of the test site.

Nevertheless, the topography of the land and the general wind regime of the zone can also lead continental winds to influence salinity values. This has been shown in a study by the Academy of Sciences in Russia [65,68] involving long-term studies at Murmansk and Vladivostok, which concluded that chloride entrainment in both areas was dependent on both the average speed of total winds (marine + continental) and the product of the wind speed by its duration (wind power).

Further studies on this effect would be of great interest as a fuller knowledge would allow, for instance, the estimation of atmospheric salinities simply by analysing information on winds available in existing meteorological databases. The inclusion of salinity values in the numerous published damage functions between corrosion and environmental factors would also make it possible to estimate MAC at a specific site from meteorological data without the need to carry out lengthy and expensive natural corrosion tests.

### 4.4. Effect of Salinity on Steel Corrosion

For salt to accelerate corrosion the metallic surface must be wet. Preston and Sanyal [69] showed that corrosion of an iron surface under a deposit of NaCl particles starts to be seen at 70% RH, and is notably accelerated at higher RH. However, Evans and Taylor also note that sea salt particles cause corrosion at a lower RH than NaCl particles, due to the fact that sea salt contains very hygroscopic magnesium salts [70].

#### 4.4.1. Steel Corrosion versus Salinity

**Figure 7:**
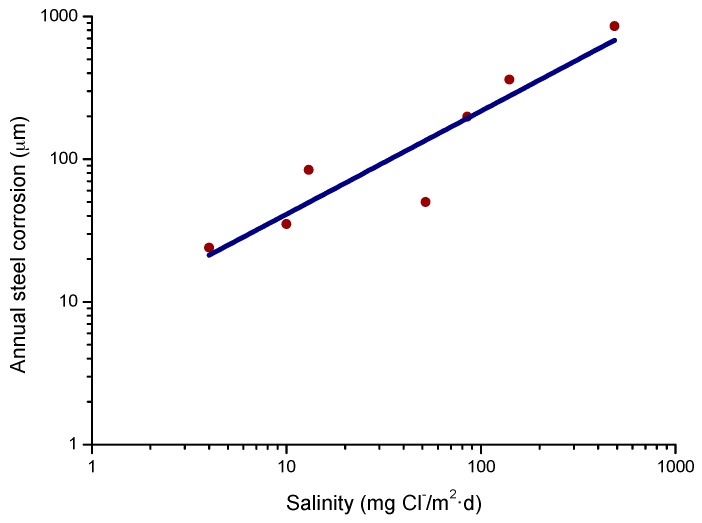
Annual steel corrosion versus salinity according to Ambler and Bain [9].

**Figure 8:**
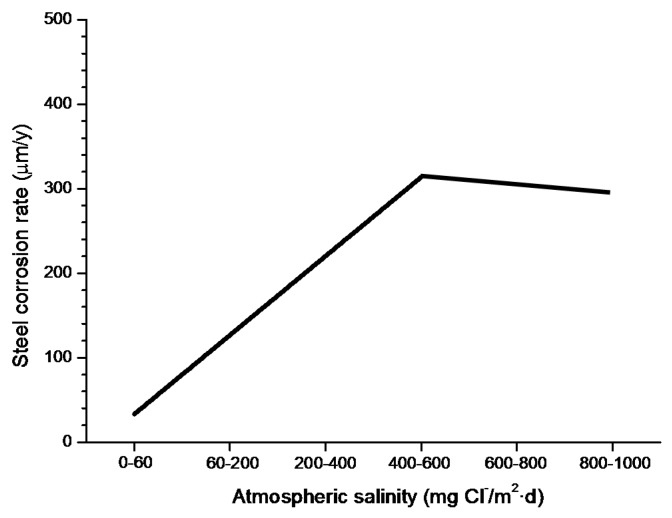
Variation in the corrosion rate of mild steel with salinity over a broad spectrum of atmospheric salinities. The graph shows a trend. Information obtained in an exhaustive bibliographic search [64].

In studies of MAC a direct relationship is generally established between corrosion and the saline content of the atmosphere. Ambler and Bain were the first to demonstrate this relationship [9]. In Figure 7, corresponding to the studies of Ambler and Bain [9], it is clearly seen how steel corrosion already experiences a notable acceleration at low atmospheric salinities, increasing from 10 to 100 mg Cl^−^/m^2^·d, as has also been reported by many other researchers. This effect has subsequently been addressed in other papers [64,71]. Figure 8 shows the variation in the CS corrosion rate with atmospheric salinity over a broad spectrum of airborne salt concentrations [64].

For salinities of less than 600 mg Cl^−^/m^2^·d, a linear relationship trend between both parameters can be deduced, with the CS corrosion rate increasing considerably as the atmospheric salinity rises. For salinities above this value the corrosion rate seems to be stabilised.

Only a small number of MAC studies have been carried out at sites with very high atmospheric salinities. Morcillo et al. observed less steel corrosion at sites with a high Cl^−^ deposition rate (1905 mg/m^2^·d) than at other very nearest site with lower atmospheric salinity values (824 mg/m^2^·d) [12]. The explanation of this fact lies in the lower oxygen solubility in the aqueous layer on the metallic surface at a very high Cl^−^ concentration. Oxygen is a fundamental element for the cathodic process of metallic corrosion. This finding is not an isolated occurrence. Pascual Marqui [72] explains this effect in terms of competitive adsorption: at high Cl^−^ concentrations the adsorbed O_2_ concentration on the metal surface is lower, in contrast to the adsorption of Cl^−^ ions. Espada et al. [73] also observed this effect with salt fogs at high NaCl concentrations. In another study by Hache [74] it was experimentally seen in immersion tests that both steel corrosion and dissolved oxygen decreased when the saline solution concentration exceeded a threshold of 10 g NaCl/L.

The literature contains very little steel corrosion data corresponding to salinities above 600 mg Cl^−^/m^2^·d. It would be important to have more information from very severe marine atmospheres in order to rigorously confirm these observations.

#### 4.4.2. Steel Corrosion versus Distance from the Shore

The influence of the distance from the sea is one of the most important aspects of MAC in coastal areas. Empirically, it is known that the effect of marine atmospheres basically runs to a few hundred metres from the shoreline and decays rapidly further inland.

The complexity of the phenomena associated with MAC makes it difficult to devise a model that can cover all possible scenarios. However, for areas closest to the shoreline (~400 to 600 m), published data shows that the decrease in the corrosion rate with the distance from the sea is fairly well represented by a simple exponential relationship [60].

C = C_0_ exp(−βX) + A, (12)

where C is the corrosion rate, C_0_ is the corrosion rate at the shoreline; β is a constant; X is the distance inland from the shoreline; and A is the corrosion rate at zero salinity.

**Figure 9:**
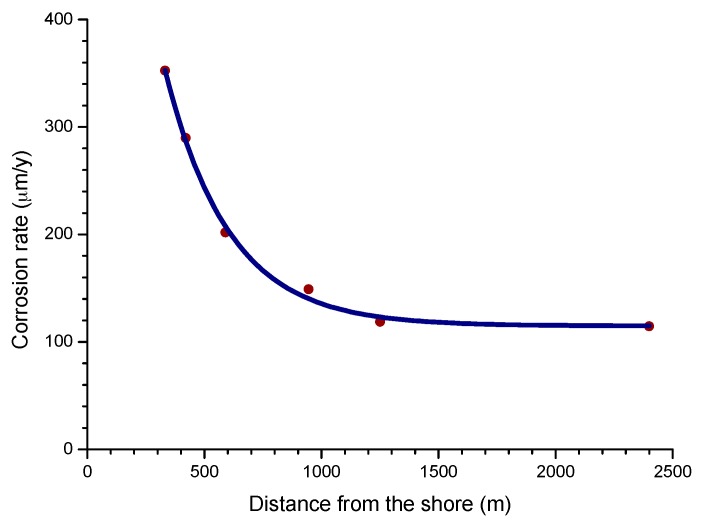
Variation in the corrosion rate of mild steel with distance from the shore [64].

Bearing in mind the aforementioned relationship between steel corrosion and atmospheric salinity, the variation in corrosion with the distance from the shore (Figure 9) should be an exponential function similar to that observed in Figure 5 between this variable and atmospheric salinity.

### 4.5. Measurement of Atmospheric Salinity

Airborne salinity is the amount of marine aerosol present in a given marine atmosphere, and a value that is commonly measured in corrosion studies. Strekalov carried out an important review of this matter [75].

In MAC studies chlorides are usually captured by the wet candle method [9] or the dry cloth (or gauze) method [76,77], both of which are set out in ISO standard 9225 [78]. The dry cloth method was developed in the former Soviet Union and is also widely used in Asia. Both methods offer great benefits from the point of view of corrosion studies as they are suitable for long-term measurements (usually one month in duration) and the fact that their data refers to the amount of salt deposited per unit of surface area (generally expressed as mg Cl^−^/m^2^·d), which is a more relevant indicator for the corrosion process than the saline content per unit of air volume. Foran et al. [79] suggest the possibility of measuring atmospheric salinity simply by determining the amount of chlorides dissolved in the rainwater collected in pluviometers.

As is noted in ISO 9223 standard [30], the results obtained by applying these various methods are not always directly comparable or convertible. In fact, ISO 9225 standard [78] provides a number of conversion factors. Corvo et al. [80] find that the following relationship:

[Cl^−^]_wc_ (mg/m^2^·d) = −54.5 + 1.6 [Cl^−^]_dc_ (mg/m^2^·d), (13)

where:

[Cl^−^]_wc_ = salinity determined by the wet candle method
[Cl^−^]_dc_ = salinity determined by the dry cloth method
Is only valid for salinity values of a considerable magnitude.

**Figure 10:**
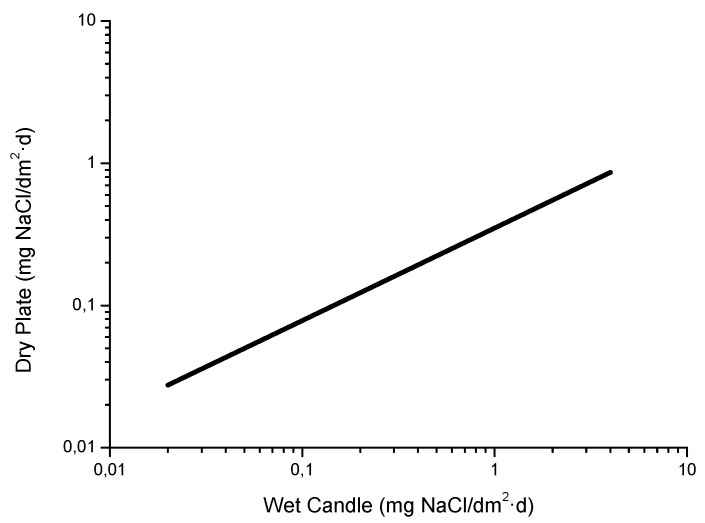
Relationship between salinity values reported by wet candle and dry plate methods [81].

Figure 10 shows the relationship between the Cl^−^ deposition rates measured using both methods at two sites in Japan [81]. It is seen that the wet candle method is more sensitive to the presence of NaCl, capturing a greater amount of aerosol than the gauze method for NaCl levels of more than 5 mg/m^2^·d.

In Australia, the INGALV Corrosion Mapping System [82] appears to calculate the Cl^−^ deposition rate at any location primarily on the basis of its proximity to the coast. However, it may be misleading to rely on simple subjective appreciations in the hope of correlating environment and pollution. For instance, points relatively close to the shoreline may in fact have lower Cl^−^ levels that a simple glance at the area and its surroundings might seem to suggest.

Finally, the Civil Research Institute (CRI) of the Ministry of Construction in Japan developed the CRI method in order to allow the absorption of a larger amount of salt than the relatively limited gauze method (Japanese standard JIS-Z-2381 [83]). This method uses a large capacity salt collector [84].

### 4.6. Salt Lake Atmospheres

Very little research work has focused on steel corrosion in salt lake environments, though several papers have recently been published in relation with Qinghai salt lake in north-west China [85,86]. Qinghai salt lake possesses an extremely high Cl^−^ ion concentration, with an average of 34 wt % Cl^−^ ions in the salt lake water, ten times that of seawater. It is important to note that the Mg content in the salt water of this lake is very high, much higher than other cations in the salt lake water and in seawater, and must be taken into account that the critical RH for MgCl_2_ is 35%, much lower than the 75% corresponding to NaCl.

According to the authors, as the steel surface went though wet/dry cycles the alien magnesium cations got a good chance to participate in the corrosion reactions by replacing the ferrous ions and forming Mg-containing intermediates.

### 4.7. Deicing Salts

Although typically associated with marine environments, NaCl is actually more prevalent in the environment from the use of road deicing salt. Extensive use of deicing salts for snow removal, generally NaCl with small amounts of CaCl_2_ and MgCl_2_, began in the early 1960s. Heavy use of deicing salt, as much as 20 tons per lane mile per year, is common throughout regions of the snow belt in the northern states of the US. The widespread use of salt has been associated with a significant amount of damage to the environment and highway structures. Road spray, dirt and salts are carried by the air blast created by heavy traffic and quickly contaminate horizontal specimens. The prolonged wet period caused by deposits, chlorides and sulfates in close contact with the steel tends to accelerate poultice corrosion [42,87].

It is well known that deicing salts often cause corrosion problems and produce thick and flaky rust on steel bridges. This kind of rust is strongly dependent upon the local environment and topography around the bridges, where the RH is usually high, the air circulation is poor, and the steels are exposed to wetness for long times. In addition, chlorides accumulate in the rusts on girders that receive less washing from rainfall. Cook et al. [42] have evaluated several WS bridges in the USA exposed to road deicing salt and showing signs of significant corrosion and exfoliated rust. Rust samples have been collected from steel girders directly above roadways that are regularly deiced during winter. In these locations total thickness losses of about 1.5 mm have been measured on the girders over a period of 20 years.

Takebe et al. [88] estimated the amount of Cl^−^ from deicing salts on WS used for bridges and developed a method to evaluate the amount of salt present on bridge girders due to deicing salts. The sampling method is described in [89].

In a review of publications on this matter in the USA and Japan, Hara et al. [90] reported that Cl^−^ concentrations exceeding approximately 0.2–0.3 wt % in the rusts accelerated the increase in rust thickness and led to the development of extremely thick rust layers. A countermeasure to this problem is to periodically wash adhered salts from the girders. According to Hara et al. [90], periodic washing with pressurised tap water, delivering 2–4 MPa (high pressure washing) at the outlet nozzle, effectively suppresses the growth of rust particles by Cl^−^ ions and the development of thick rust layers, and may be useful as a suppression technology for deicing salts.

## 5. Atmospheric Corrosion Products

Atmospheric corrosion products of iron, referred to as rust, comprise various types of oxides, hydroxides, oxyhydroxides and miscellaneous crystalline and amorphous substances (chlorides, sulfates, nitrates, carbonates, etc.) that form as a result of the reaction between iron and the atmosphere [91] (Table 1).

In marine environments other rust products not listed in Table 1 may also appear, in some cases quite significantly. These include ferrous and ferric chlorides (FeCl_2_ and FeCl_3_), ferrous-ferric chloride [Fe_4_Cl_2_(OH)_7_], etc., which are highly stable and therefore easily leachable from the corrosion product layers during atmospheric exposure. Table 2 shows the iron corrosion species that contain chlorine in their composition. Gilberg and Seeley [92] have investigated the context in which Cl^−^ ions can be found within iron corrosion products. Thus, they note that FeCl_3_ and FeOCl are unstable to hydrolysis, being converted to akaganeite.

After short-term atmospheric exposure, oxyhydroxides (lepidocrocite, goethite, and akaganeite) and oxides (magnetite and maghemite) are the main crystalline products comprising the rust layers. The composition of the rust layer depends on the conditions in the aqueous adlayer and thus varies according to the type of atmosphere.

One matter that has not yet been completely clarified is the content and composition of the amorphous phase of rust. Authors often try to study the structure of the corrosion products by quantitative powder XRD. The amorphous phase represents the difference between the sum of all the crystallised phase portions and 100%. According to Dillmann et al. [47], because powder XRD quantitative measurements are not very precise (about 10–20 relative percent error), measurements of the amorphous part of rust provided by this method need to be considered with great caution. If the techniques normally used to identify the different crystalline phases of rust (XRD, Fourier transform infrared (FTIR), MS, Raman spectroscopy (RS)) often have difficulty discriminating one phase from another, in the case of less crystallised (amorphous) rust phases such as feroxyhyte (δ-FeOOH), ferrihydrite, etc. this difficulty is further exacerbated. Monnier et al. [93] and Neff et al. [94] suggest combining the use of different complementary techniques in order to obtain improved characterisation, e.g., using µXRD, X-ray absorption under synchrotron radiation, µRS, etc.

It is unanimously accepted that lepidocrocite (γ-FeOOH) is the primary crystalline corrosion product formed in the atmosphere. In marine atmospheres, where the surface electrolyte contains chlorides, akaganeite (β-FeOOH) is also formed. As the exposure time increases and the rust layer becomes thicker, the active lepidocrocite is partially transformed into goethite (α-FeOOH) and spinel (magnetite (Fe_3_O_4_)/maghemite (γ-Fe_2_O_3_)). An increase in the airborne Cl^−^ deposition rate is accompanied by a drop in the lepidocrocite content of the rust and a rise in the goethite, akaganeite and spinel contents [95], as will be seen later.

Bernal et al. [96] in 1959 identified the conditions for the formation of the different iron oxides and hydroxides, noting the importance of the physicochemical conditions of the aqueous adlayer on the oxidation products of Fe(OH)_2_: goethite, feroxyhyte, green rusts, spinels, etc. They also noted that the common feature of the group of iron oxides and hydroxides was that they were composed of different stackings of close-packed oxygen/hydroxyl sheets, with various arrangements of the iron ions in the octahedral or tetrahedral interstices.

### 5.1. Most Significant Corrosion Products in Steel Corrosion in Marine Atmospheres

The following section focuses on the most significant corrosion products of steel when exposed in Cl^−^-rich atmospheres, describing their formation mechanisms, structure, etc.

#### 5.1.1. Green Rust 1 (GR1 or GR(Cl^−^))

Green rusts (GR) are unstable intermediate products, very often amorphous, which occasionally emerge in the presence of anions such as Cl^−^, SO_4_^2−^, etc., and replace OH^−^ ions in processes involving the ferrous-ferric transformation of hydroxides, oxides and oxyhydroxides in poorly aerated environments. Their name is derived from their bluish-green colour [97]. Two broad groups of GR have been distinguished. One contains primarily monovalent anions such as OH^−^ and Cl^−^ and is designated GR1, while the other contains mainly divalent ions such as SO_4_^2−^ and is designated GR2 [98].

Green rusts rarely exhibit a well-defined stoichiometry and their composition depends on the particular environmental conditions. The formula of GR1 sometimes reported in the literature is [3Fe(OH)_2_·Fe(OH)_2_Cl·nH_2_O], containing an equal number of Cl^−^ and Fe^3+^ ions, while GR2 conforms to the formula [2Fe(OH)_3_·4Fe(OH)_2_·FeSO_4_·nH_2_O] [99].

The crystal structures of GRs are assumed to be similar to that of the mineral pyroaurite [100], Mg_6_^II^Fe_2_^III^(OH)_16_CO_3_·4H_2_O. According to Refait et al. [99] a structural model derived from the pyroaurite structure can be reasonably proposed for GR1. The Fe atoms of the hydroxide layers are randomly distributed among the octahedral positions. The interlayers are mainly composed of Cl^−^ ions and O_2_ atoms belonging to the water molecules connecting two OH^−^ ions of adjacent hydroxide layers.

GR1 is usually prepared by aerial oxidation of Fe(OH)_2_ suspensions in the presence of a slight excess of dissolved FeCl_2_. Thus, in slightly basic and Cl^−^-containing aqueous media, GR1 should be obtained as a corrosion product of iron and steels either by oxidation of an initial Fe(OH)_2_ layer or by direct precipitation in the simultaneous presence of Fe^2+^ and Fe^3+^ dissolved species.

7Fe(OH)_2_ + Fe^2+^ + 2Cl^−^ + ½O_2_ + (2n + 1)H_2_O→2[3Fe(OH)_2_·Fe(OH)_2_Cl·nH_2_O], (14)

GR1 found in Cl^−^-containing aqueous media occurs during the corrosion of steels before the formation of the end products such as lepidocrocite, goethite, akaganeite and magnetite, as its formation is more favoured [99].

#### 5.1.2. Akaganeite ((β-FeOOH) or β-FeO(OH,Cl^−^))

Akaganeite is the rust phase of capital importance in the MAC process of steel, and thus is discussed here in the greatest detail. Akaganeite is one of the polymorphs of ferric oxyhydroxides (-FeOOH). Its formation requires halogen ions to stabilise its crystalline structure. Since it always contains Cl^−^ ions, this compound is not strictly speaking an oxyhydroxide. Stahl et al. have determined its chemical formula as FeO_0.833_(OH)_1.167_Cl_0.167_ [101].

Watson et al. [102] observed that the crystal possessed a regular porous structure and suggested that the subcrystals might not be solid rods but tubes which, though externally still square prisms, contained a circular central channel or tunnel running the whole length of the subcrystal. The tunnels in the akaganeite structure, with a diameter of 0.21–0.24 nm, are stabilised by Cl^−^ ions, and Cl^−^ levels ranging from 2 to 7 mol % have been reported. A minimum amount of Cl^−^, 0.25–0.50 mmol/mol seems essential to stabilise the crystalline structure of akaganeite [91]. According to Keller [103], akaganeite has been shown to contain up to 5 wt % Cl^−^ ions in marine atmospheres. At ambient temperature these tunnels are full of water and Cl^−^ [91]. The impossibility of leaching the Cl^−^ by washing confirms that at least part of the Cl^−^ ions are found in the crystalline lattice, as noted by Rezel et al. [104] and Ståhl et al. [101].

Gallagher [105] describes akaganeite as a fascinating substance that precipitates as unusual cigar-shaped crystals with a tetragonal unit cell, although this has given rise to much controversy. The structural refinement by XRD (Rietveld) carried out by Post and Buchwald confirms that the unit cell is monoclinic, having eight formula units per unit cell [106]. The Fe^3+^ ions were each surrounded octahedrally by six OH^−^ ions.

The crystals are very small and the crystallographic structure is isostructural with hollandite (BaMn_8_O_16_) characterised by the presence of tunnels parallel to the C-axis of the lattice. The size distribution of the crystals is fairly narrow and their length is only exceptionally greater than 500 nm. Due to its special structure (presence of tunnels) akaganeite is less dense than other oxyhydroxides like lepidocrocite or goethite [107,108]. In this respect, Shiotani et al. note that akaganeite has a relatively larger volume in relation to the initial iron [109].

Akaganeite displays two basic morphologies: somatoids (spindle-shaped crystals) and rods (cigar-shaped crystals). The former type is the usual morphology of akaganeite when it forms in laboratory conditions by hydrolysis of acid FeCl_3_ solutions at 25–100 °C [107,108]. The latter type, according to the authors’ experience, is the usual morphology of the akaganeite crystals that form in atmospheric conditions. Researchers have assigned SEM morphologies to akaganeite without an unequivocal characterisation of this oxyhydroxide. Morcillo et al. were able to do this using the SEM/µRS technique [110,111], observing aggregates of akaganeite crystals, a sponge-type morphology, constituted by a lattice of elongated cylinder- or tube-shaped crystals typical of the rod morphology (cigar-shaped crystals) of this oxyhydroxide.

With regard to akaganeite formation mechanisms when steel is exposed to a Cl^−^-rich marine atmosphere, the following may be noted. The formation of akaganeite is preceded by the accumulation of Cl^−^ ions in the aqueous adlayer giving rise to the formation of FeCl_2_, which hydrolyses water according to:

FeCl_2_ + 2H_2_O→Fe(OH)_2_ + 2HCl, (15)

At the steel/corrosion products interface, where Cl^−^ ions accumulate, high Cl^−^ concentrations and acidic conditions with pH values between 4 and 6 give rise to the formation of ferrous hydroxychloride (β-Fe_2_(OH)_3_Cl), a very slow process requiring the transformation of metastable precursors [112,113]. Remazeilles and Refait concluded that large amounts of dissolved Fe(II) species and high Cl^−^ concentrations are both necessary for akaganeite formation [114]. The oxidation process of β-Fe_2_(OH)_3_Cl which leads to akaganeite formation passes through different steps via the formation of intermediate GR1 (Fe_3_^II^Fe^III^(OH)_8_Cl^−^nH_2_O) [99,113,115]. In all, the whole oxidation process leading to akaganeite can be summarised as follows [99,112,113,114,115]:

FeCl_2_→β-Fe_2_(OH)_3_Cl→GR1 (Cl^−^)→β-FeOOH, (16)

Thus requiring a relatively long time.

#### 5.1.3. Magnetite (Fe_3_O_4_)/Maghemite (γ-Fe_2_O_3_)

The structure of magnetite is that of an inverse spinel. Magnetite has a face-centred cubic unit cell based on thirty-two O^2−^ ions which are regularly cubic close packed. There are eight formula units per unit cell [91]. Magnetite differs from most other iron oxides in that it contains both divalent and trivalent iron. Its formula is written as Y[XY]O_4_, where X = Fe^II^, Y = Fe^III^ and the brackets denote octahedral sites. Eight tetrahedral sites are distributed between Fe^II^ and Fe^III^, i.e., the trivalent ions occupy both tetrahedral and octahedral sites. The structure consists of octahedral and mixed tetrahedral/octahedral layers [91]. However, magnetite, if it were the normal spinel structure, would have eight tetrahedral sites occupied by eight Fe^2+^ ions and sixteen octahedral sites occupied by sixteen Fe^3+^ ions [116].

In stoichiometric magnetite Fe^II^/Fe^III^ = 0.5, however, magnetite is frequently non-stoichiometric, in which case it has a cation-deficient Fe^III^ sub-lattice. Magnetite is also said to have a defect structure with a narrow composition range, the Fe:O ratio of which varies from 0.750 to 0.744 [117]. Thus, magnetite usually presents vacancies, preferably on octahedral sites, which form to maintain the electroneutrality of the crystal when H_2_O or OH^−^ molecules enter the network, as well as ferrous and ferric ions sharing their valence electrons.

Maghemite has a similar structure to magnetite, but differs in that all or most Fe is in the trivalent state. Cation vacancies compensate for the oxidation of Fe^II^. Maghemite also has a cubic unit cell, each cell contains thirty-two O^2−^ ions, twenty-one and one-third Fe^III^ ions and two and a third vacancies. Eight cations occupy tetrahedral sites and the remaining cations are randomly distributed over the octahedral sites. The vacancies are confined to the octahedral sites [91]. Maghemite is also a defect structure with the Fe:O ratio in the range of 0.67–0.72 [117].

XRD presents an important limitation when it comes to differentiating the magnetite phase from the maghemite phase, as both show practically identical diffractograms (similar crystalline structures) and are very hard to differentiate when mixed with large amounts of other phases (lepidocrocite, goethite and akaganeite), as occurs in the corrosion products formed on steel when exposed to marine atmospheres. Both phases are associated to the diffraction angle at 35° [118]. Both phases are usually detected in the inner part of the rust adhering to the steel surface, where oxygen depletion can occur [119,120].

Spinel phase (magnetite and/or maghemite) may form by oxidation of Fe(OH)_2_ or intermediate ferrous-ferric species such as green rust [119]. It may also be formed by lepidocrocite reduction in the presence of a limited oxygen supply [120] according to:

2γ-FeOOH + Fe^2+^→Fe_3_O_4_ + 2H^+^, (17)

With a broader view, Ishikawa et al. [121] and Tanaka et al. [122] found that the formation of magnetite particles was caused by the reaction of dissolved ferric species of oxyhydroxides with ferrous species in the solution, in the following order: akaganeite > lepidocrocite >> goethite. The formation of magnetite can be represented as the following reaction:

Fe^2+^ + 8FeOOH + 2e^−^→3Fe_3_O_4_ + 4H_2_O, (18)

Remazeilles and Refait [114], Nishimura et al. [10] and Lair et al. [40] all observe that the electrochemical reduction of oxyhydroxides leads to spinel phase formation.

As Hiller noted some time ago, the rust formed in marine atmospheres contains more magnetite than that formed in Cl^−^-free atmospheres [123]. In severe marine atmospheres the spinel phase can be the main rust constituent, as was found by Jeffrey and Melchers [124] and by Haces et al. [125].

There is often uncertainty as to which of the two phases, magnetite or maghemite, is present in AC products, and indeed both species could be present depending on the local formation conditions and the corrosion mechanisms involved in the process. This lack of definition may also be intimately related with the analytical techniques used for their determination. Many researchers have reported the presence of magnetite in AC products on the basis of XRD data, but much of this data is suspect since the XRD patterns of magnetite and maghemite are very similar. The same happens when the ED method is used [126]. However, Graham and Cohen [127] do show convincing evidence on the basis of MS that magnetite is a component of corrosion products on several samples. However, Leidheiser and Music [128] and Chico et al. [129], also using this technique, found no evidence of magnetite. Likewise, Oh et al. [130], using MS and RS, find a high magnetic maghemite content in the exposure of CS at 250 m from the seashore. In contrast, Nishimura et al. [10], using X-ray photoelectron spectroscopy (XPS) and TEM, find high magnetite contents.

Antony et al. [131] reported that FTIR is also not very appropriate to precisely identify magnetite, and Monnier et al. [93] also note that MS has difficulty in discriminating phases of the same oxidation state that have similar local environments, particularly in the case of complex mixes, as is the case of magnetite and maghemite. Thus, it seems that the specific nature of each analysis method strongly influences the type of phase identified.

The identification of rust amorphous phases as well as the classification of the type of spinel formed (magnetite or maghemite) are two issues where more research effort is needed.

### 5.2. Other Characteristics of the Steel Atmospheric Corrosion Products

#### 5.2.1. Towards a Greater Knowledge of the Structure of Iron Oxides

As Bernal et al. [96] suggested in 1959, the common feature of the group of iron oxides and hydroxides is that they are composed of different stackings of close-packed oxygen/hydroxyl sheets, with various arrangements of the iron ions in the octahedral or tetrahedral interstices, and their mutual transformations are topotactic by rearrangement of the atoms. These authors interpreted in a rational crystallochemical way the transformations involving the compounds Fe(OH)_2_, δ-FeOOH, FeO, γ-Fe_2_O_3_, α-FeOOH, α-Fe_2_O_3_ and Fe_3_O_4_. Only some of these transformations were not topotactic and seemed to have dissimilar structures, with renucleation being necessary for the transformation process. This is the case, for instance, with β-FeOOH→α-Fe_2_O_3_ [96].

**Figure 11:**
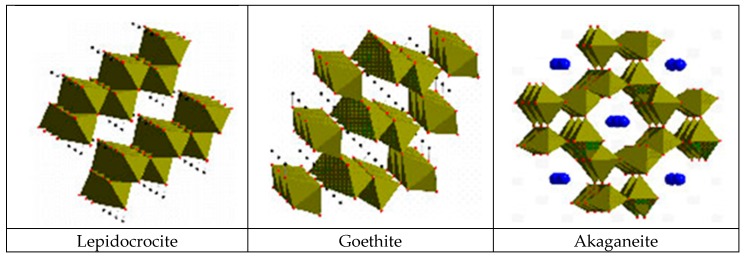
Ideal crystallographic structures of lepidocrocite (γ-FeOOH), goethite (α‑FeOOH) and akaganeite (β-FeOOH). The structures are described using FeO_6_ octahedral units. Small circles: hydrogen, medium circles: chlorine [132,133].

According to Matsubara et al. [132], it is important to know the fundamental structures of the components of iron corrosion products in order to understand the characteristic features of various types of corrosion products. The ideal crystallographic structures of three ferric oxyhydroxides—lepidocrocite, goethite and akaganeite—are described using FeO_6_ octahedral units (Figure 11). Furthermore, the structure of a Fe(OH)_2_ is composed of layers of FeO_6_ octahedra intercalated with hydroxyl OH^−^. There are also several kinds of GR containing ferric and ferrous ions which have a layered structure as Fe(OH)_2_. In the structure of GR, the fractions of ferric and ferrous ions in layers of FeO_6_ octahedra are variable and different anions and water molecules are intercalated between the layers. Although there are other iron oxide structures including hydroxides, they are fundamentally described in a similar way.

MS has been used to identify components in corrosion products and to analyse their fine structures. Other analytical methods, such as EPMA, TEM, FTIR and RS are often used for analysing corrosion products formed on the surface of steel. The results obtained by these methods provide information on the composition, morphology and structure of corrosion products. However, structural information on corrosion products obtained by these methods is limited [132].

As was seen in Section 3, in order to characterise corrosion product structures in various scales, Kimura et al. [45] have used several analytical approaches that are sensitive to three structural-correlation lengths (LRO, MRO and SRO). Thus, conventional XRD techniques can detect detailed structural information in terms of LRO. However, this technique yields broad peaks when the grain size is smaller than ~50 nm, as is often found in corrosion products. Contrarily, XFAS is useful for in-situ observation of SRO. In XAFS, oscillatory modulation near an X-ray absorption edge of a specific element of a specimen provides information in terms of the local structure around an atom (Fe, Cl, etc.) in the rust layer or determines the distance between the centred atom and the neighbouring ligands, the number of ligands, and the stereographic arrangement of ligands. However, some reservations should be made regarding information about linkage of the FeO_6_ octahedral unit structure, because XAFS data is obtained only from near neighbour atomic arrangements. This strongly suggests the great importance of middle-range ordering (MRO) for characterising corrosion products. This has been achieved by a combination of X-ray scattering (AXS) and reverse Monte Carlo simulation (RMC), which visualises the atomic configurations [45].

#### 5.2.2. Morphology

The rust formed on steel when exposed to the atmosphere is usually a complex mixture of several phases. Moreover, each of these phases can take on a wide variety of morphologies depending on their growth conditions. Thus, the diversity of rust morphologies formed on CS exposed to marine atmospheres is enormous, with a great variety of shapes and sizes of the crystalline aggregates that reflect to a large extent the different growth conditions: chemical characteristics of the aqueous adlayers formed by humidity condensation, rainfall, etc., temperature, wet/dry cycle characteristics, etc.

Cornell and Schwertmann dedicated an entire chapter of their well known and well referenced book “The iron oxides, structure, properties, occurrence and uses” to iron oxide crystal morphology and size, mainly concerned with synthetic iron oxides. Table 3 shows the principal habits (morphologies) of iron oxides according to Cornell and Schwertmann [91].

It should however be noted that the morphologies of synthetic iron oxides produced in laboratory conditions may be very different to those obtained during CS corrosion in the atmosphere, as pointed out by Waseda and Suzuki in the preface to an interesting book on “Characterisation of corrosion products on steel surfaces” [134]. As they note, the morphology of AC products is often not describable in terms of typical iron oxide structures but is much more complicated; the component phases in rust formed on steel in outdoor exposure show imperfections in their structures and real component structures appear to diverge from an ideal crystallographic structure of typical iron oxides.

For some time now, articles published on AC studies usually include SEM views of rust formations and in some cases even attribute certain morphologies to specific rust phases without an analytical characterisation. An exception can be seen in the pioneering work of Raman et al. [135]. These researchers attempted to indirectly identify the morphologies observed by SEM by comparison with the morphologies of standard rust phases grown in the laboratory and identified by XRD and IRS.

Very recently, the research group of Morcillo et al. has progressed in this field using the powerful SEM/µRS spectroscopic technique to perform a more direct and rigorous characterisation of the different morphologies that can be displayed by the main rust phases (lepidocrocite, goethite, akaganeite and magnetite) formed on CS specimens exposed to marine atmospheres for a certain time [110,111,136,137].

Without seeking to be exhaustive, there follows a tentative classification of the different types of morphology observed by the authors in the rust formed on steel exposed to marine atmospheres [137]:

(a) Globular: hemispheric-shaped aggregated formations like small mounds.
(b) Acicular: aggregates with a similar appearance to needles, hairs, or threads.
(c) Laminar: this can appear in a wide range of different formations in which laminas grow perpendicularly to the surface: bar shape, worm nest shape, bird’s nest shape, flower petal shape, feather shape, etc.
(d) Tubular: formations in which the crystalline aggregates are constituted by prisms, tubes, or rods, etc.
(e) Toroidal
(f) Geode-type: unusual or singular oolitic or globular morphology constituted by fish-egg-like spherical formations.

**Figure 12:**
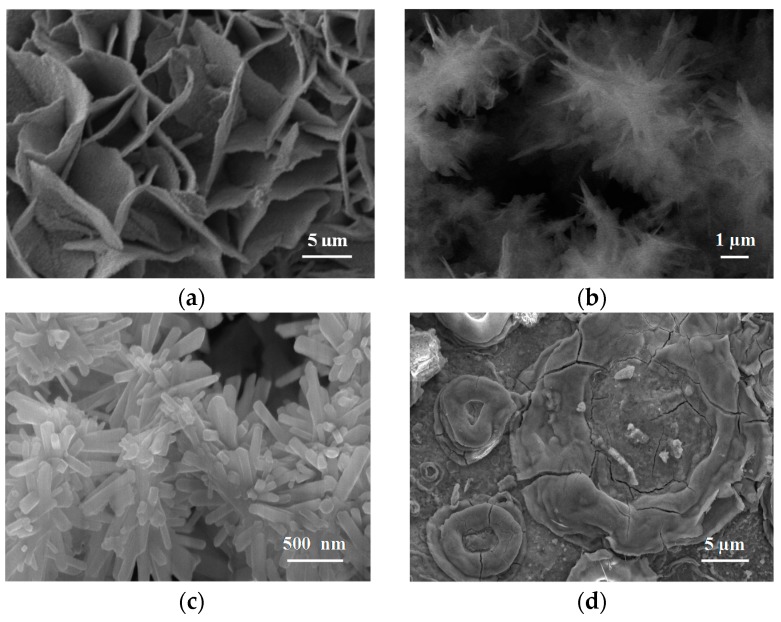
SEM view of laminar lepidocrocite (**a**); acicular goethite (**b**); tubular akaganeite (**c**) and toroidal magnetite (**d**) formations [137].

Figure 12 presents typical characteristic morphologies of the four rust phases normally present among the corrosion products formed in marine atmospheres: lepidocrocite, goethite, akaganeite and magnetite.

#### 5.2.3. Grain Size (Granulometry)

**Figure 13:**
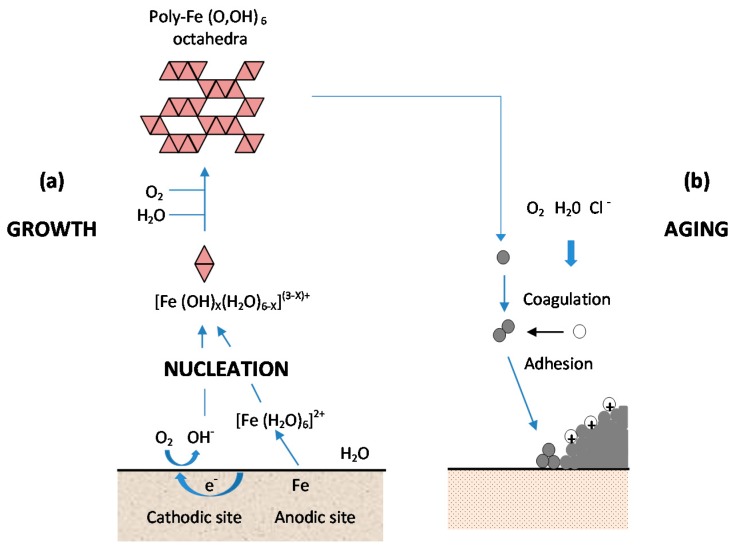
Scheme of iron corrosion in the atmosphere according Kimura et al. [45]: (**a**) reactions in the initial wet cycle; (**b**) reactions during repetition of wet/dry cycles for a long period. Triangle pairs represent Fe(O,OH)_6_ octahedra.

As shown in Figure 13, from Kimura et al. [45], which shows a schematic illustration of corrosion on an iron surface in the atmosphere, the formation process of solid particles can be visualised by three steps: (a) nucleation; (b) growth; and (c) ageing [138].

(a) Nucleation corresponds to the first step of precursor condensation and solid formation. On an atomic scale, iron forms cations that are coordinated by six water molecules [Fe(H_2_O)_6_]^2+^ [139]. In a neutral solution, metal cations react with OH^−^, O_2_, and H_2_O resulting in the formation of hydroxo cations [Fe(OH)_X_(H_2_O)_6−X_]^(3−X)+^.
(b) Then the growth process follows, where Fe(O,OH)_6_ octahedra units as cations or smaller sized growing nuclei accumulate to form larger particles. On a colloidal scale, polymerisation of these Fe(O,OH)_6_ octahedra leads to the formation of fine particles of hydroxides, oxyhydroxides or oxides.
(c) These particles grow into grains or layers through a long period of ageing processes affected by repeated wet and dry cycles. Reaction conditions (concentration, acidity, temperature, nature of anions, etc.) have a strong influence on the structural or morphological changes of poly-octahedra during corrosion. Coagulation and adhesion processes ensue to generate corrosion products, which undergo ageing processes leading the system to stability. During ageing the particles may undergo modifications such as increases in size, changes in crystal type, changes in morphology, etc. [140]. Thus, according to Ishikawa et al. [141], steel rusts can be regarded as agglomerates of colloidal nanoparticles of ferric oxyhydroxides (goethite, akaganeite and lepidocrocite), spinels (magnetite/maghemite), and poorly recrystallised iron oxides (amorphous substances). Voids of different sizes form between the fine particles in the rust layer.

Resulting from these complicated processes, corrosion products are generally classified as coarse or fine grains, both of which are composed of crystallites and inter-crystallites. The structure in the former are similar to those of ideal crystals, while in the latter the linkage of Fe(O,OH)_6_ octahedra is disordered, due to the existence of defects and/or different sizes of Fe(O,OH)_6_.

**Figure 14:**
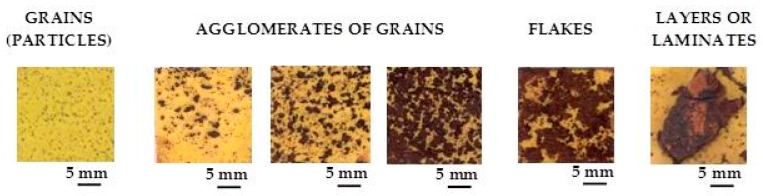
Type of rust morphologies formed on carbon steel exposed to marine atmospheres [143].

**Figure 15:**
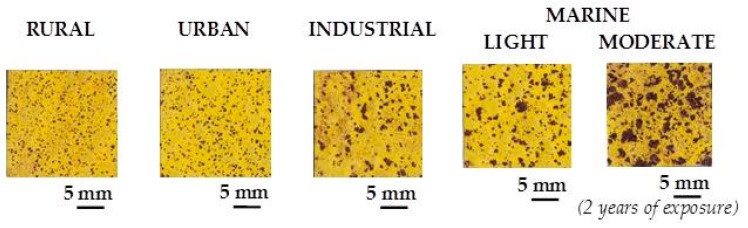
Granulometries of outermost rusts formed on skyward- facing side of carbon steel exposed for 5 years at different type of atmospheres.

**Figure 16:**
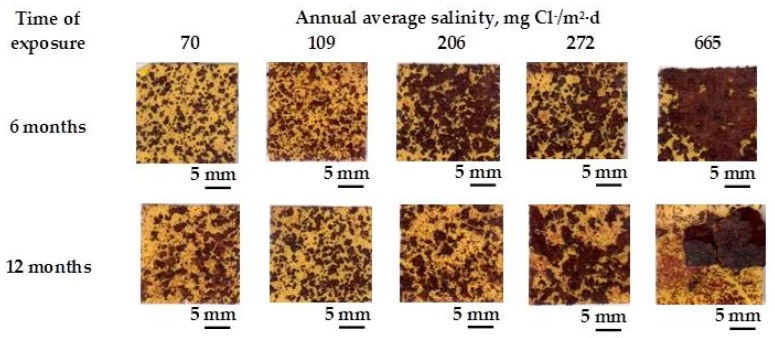
Granulometries of outermost rusts formed on skyward- facing side of carbon steel exposed for 6 and 12 months at marine atmospheres of different aggressivity [64].

A practical laboratory method for determining the grain size of rusts formed on steel during atmospheric exposure, known as the “tape method” [142], consists of adhering a 2 × 2 cm^2^ piece of adhesive tape to the outermost surface of the rust layer, pressing firmly and evenly on the surface, and lifting off to examine the size and density of rust particles. The morphology takes the form of grains or particles, agglomerates of grains, flakes, and even exfoliations (layers or laminates) [143] (Figure 14). The texture of rust is seen to vary according to the atmospheric aggressivity (Figure 15). A more heterogeneous surface appearance and coarser granulometry is found in more aggressive atmospheres (industrial and marine) [144]. In marine atmospheres, the granulometries are coarser and become more accentuated with airborne salinity and exposure time (Figure 16). In the marine atmosphere with the highest Cl^−^ deposition rate (665 mg/m^2^·d) the formation of coarse flakes and exfoliations is seen [64]. These results confirm the observations of Ishikawa et al. [144,145].

**Figure 17:**
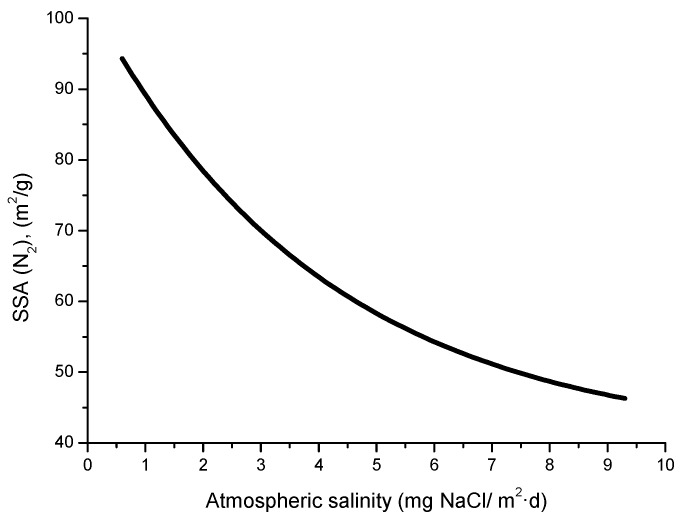
Relationships (trend curve) between SSA(N2) of rusted carbon steel and atmospheric NaCl contents [146].

The compactness of the rust layers depends on the morphology of the rust particles; smaller particles form more compact and less permeable layers. However, as Ishikawa et al. note [144], particle size analysis of rust is not easy because of the heterogeneous morphology and strong aggregation of rust particles. Ishikawa et al. [146] use the N_2_ adsorption method to estimate the particle size of rust formed on steel exposed to various situations. It was revealed that the specific surface area (SSA) obtained by N_2_ adsorption decreased with increasing airborne salinity (Figure 17). This finding shows that rust particles grow with an increase in airborne salinity, and that less compact rust layers with low corrosion resistance are formed in Cl^−^ environments such as coastal areas. In contrast, in a low salinity environment fine rust particles assemble to form densely packed rust layers with high corrosion resistance. Ishikawa et al. attribute the high SSA obtained by H_2_O adsorption on the rusts generated on the coast to the tunnels of akaganeite crystals, accessible to H_2_O but not to N_2_ [146].

Ishikawa et al., examining the texture of rusts, note that the rusts formed at coastal sites were aggomlerates of large particles and had larger pores than rusts formed at rural and urban sites. This finding suggests that NaCl promotes rust particle growth, resulting in the formation of larger pores as voids between larger particles in the rust layer and facilitating further corrosion.

## 6. The Rust Layer

When the thin layer of corrosion products has grown to cover the whole surface, further growth requires reactive species from the aqueous adlayer to be transported inwards through the rust layer while metal ions are transported outwards. In addition to this, electrons must be transported from anodic to cathodic sites on the surface, so that those produced in the anodic reaction can be consumed in the cathodic reaction. As long as the metal substrate is covered only by a thin oxide film, electron transportation through the film is generally not a rate-limiting step. However, when the corrosion products grow in thickness, electron transportation may become rate-limiting [8].

This section considers the different physical and chemical properties of corrosion product layers. It starts by addressing the organoleptical properties of rust layers, such as their colour and texture, before going on to consider other properties more related with their protective capacity: stratification, stabilisation, adhesion, thickness, and porosity and their evaluation using different indices.

### 6.1. Organoleptical Properties

#### 6.1.1. Colour

CS exposed to the atmosphere develops ochre-coloured rust which becomes dull brown as the exposure time increases. Lighter rust colours are seen in atmospheres with greater salinity (more corrosive) and darker rusts in less aggressive atmospheres [64]. In marine atmospheres, the colour of rust varies not only with the salinity of the atmosphere, but also according to the steel type, exposure time, etc.

**Figure 18:**
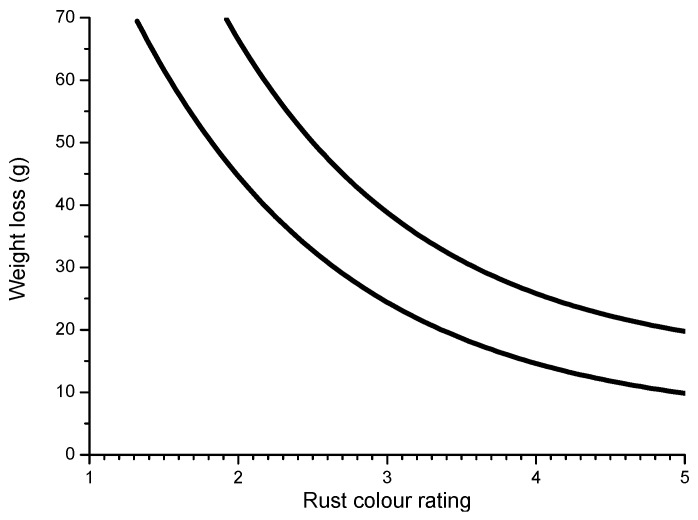
Band showing the relationships between rust colour rating after 6 months of exposure and corrosion of steels (4 × 6 in. specimens) after 7.5 years of exposure in marine atmosphere at Kure beach [147].Rust color rating: 1 (lightest)–5(darkest).

Some time ago, LaQue [147] exposed different steels for 6 months to the marine atmosphere of Kure Beach (250 m from the shoreline) and found that the colouring of 84% of the tested steels was within the range seen in Figure 18, which shows the relationship between the rust colour ratings as developed early in the test and the corrosion resistance of the steels after long-term exposure.

#### 6.1.2. Texture

In Section 5.2.3 reference was made to the granulometry of corrosion products. In part, this property is closely related with the texture of the outer surface of the rust layer. Sense of touch is used to determine aspects of texture such as smoothness, unevenness and roughness. Ph. Doctoral Thesis of I. Díaz [148] and H. Cano [149] reported one to three-year exposure of a variety of CS in different types of atmospheres, where differences in texture were observed in the rust layers formed. Patinas with smoother textures (more homogenous appearance and finer granulometry) were found in rural and urban atmospheres, while rougher textures with a more heterogeneous appearance and a coarser granulometry were observed in industrial and marine atmospheres, all the more so the higher the corrosivity of the atmosphere (higher Cl^−^ deposition rate) and the longer the exposure time.

### 6.2. Properties More Related with the Protective Capacity of Rust Layers

#### 6.2.1. Stratification of Rust Layers

**Figure 19:**
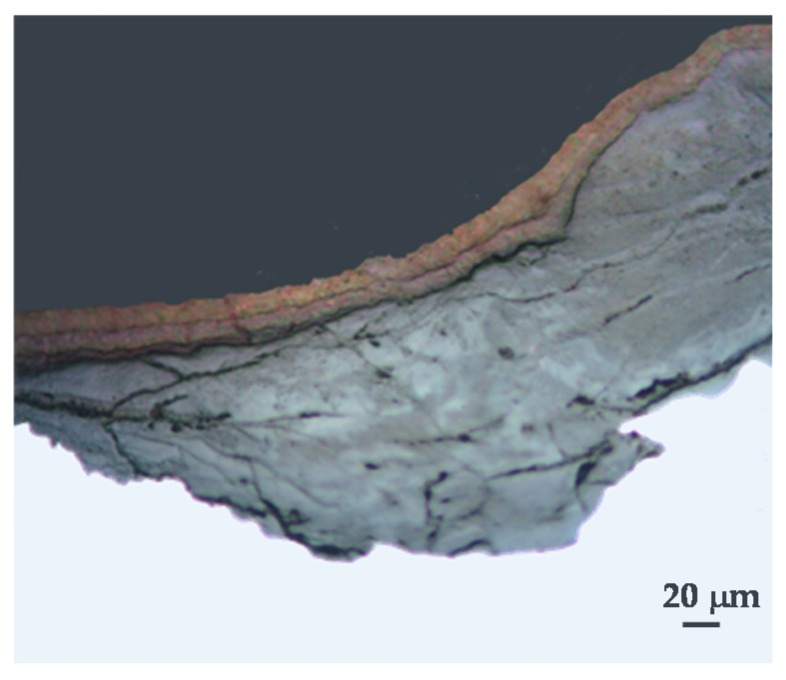
Dual structure of a consolidated rust layer formed during one year on carbon steel exposed al unsheltered conditions in a marine atmosphere with low chloride deposit (21 mg Cl^−^/m^2^·d). Optical micrograph obtained by polarized light. The outer orange-coloured layer is mainly lepidocrocite while the inner greyish layer is mainly goethite and magnetite [150].

There is controversy about the stratification of the rust layer in different sublayers on unalloyed CS [97]. According to Díaz et al. [150] rust is always stratified irrespective of the steel composition, be it WS or plain CS. In their investigations these authors have found the presence of two sublayers in all rust films: an uncoloured (dark grey) inner layer and an orangey-brown-coloured outer layer (Figure 19). Thus, the dual nature of the rust layer is not an exclusive characteristic of WS since plain CS with less AC resistance also generates a stratified rust.

According to Suzuki [151], rust layers usually present considerable porosity, spallation, and cracking. Cracked and non-protective oxide layers allow corrosive species easy access to the metallic substrate, and is the typical situation in atmospheres of high aggressivity. However, compact oxide layers formed in atmospheres of low aggressivity favour the protection of the metallic substrate. The higher the Cl^−^ deposition rate in marine atmospheres, the greater the degree of flaking observed, with loosely adherent flaky rust favouring rust film breakdown (detachment, spalling) and the initiation of fresh attack. As time elapses, the number and size of defects may decrease due to compaction, agglomeration, etc. of the rust layer, thereby lowering the corrosion rate [152,153].

#### 6.2.2. Stabilisation of Rust Layers and Steady-State Corrosion Rate

Bibliographic information on this aspect is highly erratic and variable. The gradual development of a corrosion layer takes several years before steady-state conditions are obtained, though the exact time taken to reach a steady state of AC will obviously depend on the environmental conditions of the atmosphere where the steel is exposed.

Morcillo et al. have determined the stabilisation times of rust layers formed on WS [87], considering the steady state corrosion rate to be the rate corresponding to the year from which corrosion slows by ≤10%. Previously it was confirmed that the corrosion rate (y) plotted against the exposure time (x) fitted an exponential decrease equation:

y = A_1_ exp(−x/t_1_) + y_0_, (19)

where

y = corrosion rate, µm/y
x = time, years
1/t_1_ = decrease constant
y_0_ = steady state corrosion rate, µm/y
A_1_ + y_0_ = corrosion rate at x = 0, µm/y

**Figure 20:**
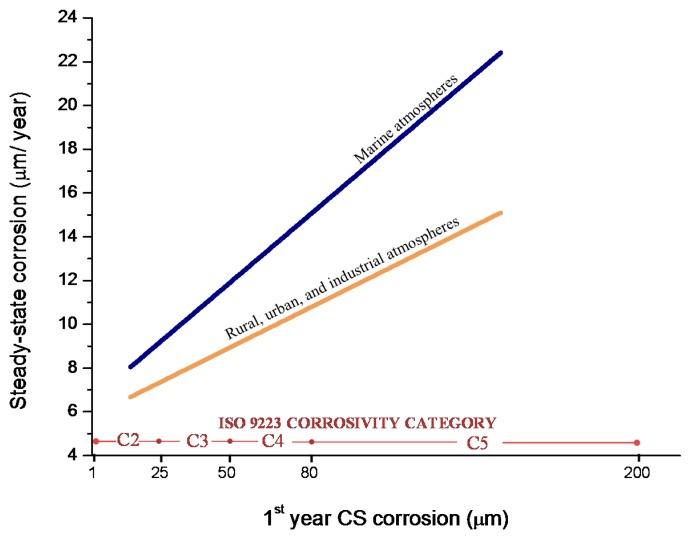
Relationship between steady-state corrosion rate of carbon steel and atmospheric corrosivity category according ISO 9223 for different type of atmospheres [87].

The rust layer stabilisation time decreases as the corrosivity category (ISO 9223) [30] of the atmosphere rises. The stabilisation time depends, among other factors, on the exposure time, the existence of wet/dry cycles, the corrosivity of the atmosphere, and in short on the volume of corrosion products formed. However, a shorter stabilisation time does not imply a greater protective capability of the rust. In this respect, stabilisation of the rust layer occurs faster in marine atmospheres, due to their greater corrosivity, but the protective value of this rust is lower than that of rusts formed in less aggressive atmospheres (rural, urban, etc.) where stabilisation times are longer. The steady-state corrosion rate increases in line with the corrosivity of the atmosphere in both rural, urban, industrial, and marine atmospheres (Figure 20) [87].

#### 6.2.3. Adhesion

According to Honzak [154] it is possible to differentiate three layers in rust: surface rust that is easily removable (e.g., by light scraping), an intermediate rust layer that can be “burst off” by bending the specimen, and a very adherent layer on the metal surface which cannot be removed by scraping but is removable by abrasive cleaning or chemical methods (e.g., pickling).

Not all steel corrosion products become incorporated in the rust layer. Some examples [124] include:

(a) Brown stains as a consequence of run-off processes
(b) Leaching of soluble components of the rust layer (iron chlorides in marine atmosphere) by rainwater, and
(c) Rust lost through abrasion and erosion, the latter particularly in high wind areas.

It has long been known that not all the corroded metal becomes part of the measurable rust product [155], but there have been very few attempts to quantify this part. One exception is the work of García et al. [156,157,158], who classified adherent rust as: (i) removable by scraping the steel surface with a metallic brush; and (ii) removable by hitting the steel with a hammer. According to these authors, the protective properties of rust on CS in a given corrosive environment depends on the characteristics of the adherent rust, i.e., that which is bonded to the metal surface, but a full overview of the corrosion process also requires the characterisation of non-adherent rust, i.e., that which is loosely bound to the metal surface, and that which is lost during the corrosion process. The authors found that the amount of corroded iron that is converted into adherent rust on steels exposed to Cl^−^ in wet/dry cycles ranged from 0.55 to 0.90, the amount of corrode iron converted into non-adherent rust ranged between 0.3 and 0.18, and the amount of iron that is lost ranged between 0.2 and 0.38.

#### 6.2.4. Thickness and Internal Structure

**Figure 21:**
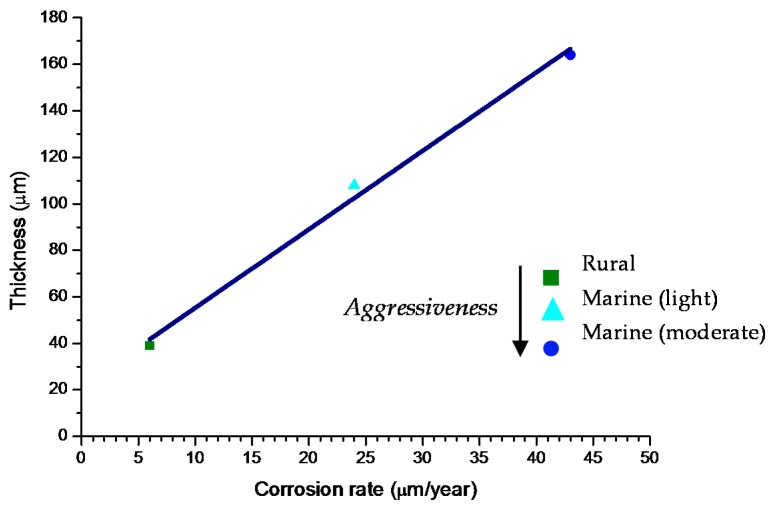
Corrosion rate versus rust thickness for low carbon steel exposed during two years in atmospheres of different aggressivity [159].

The thickness of the rust layer increases with time of exposure and the aggressivity of the atmosphere. A direct (linear) relationship is found between the rust layer thickness and the substrate corrosion rate [159] (Figure 21).

**Figure 22:**
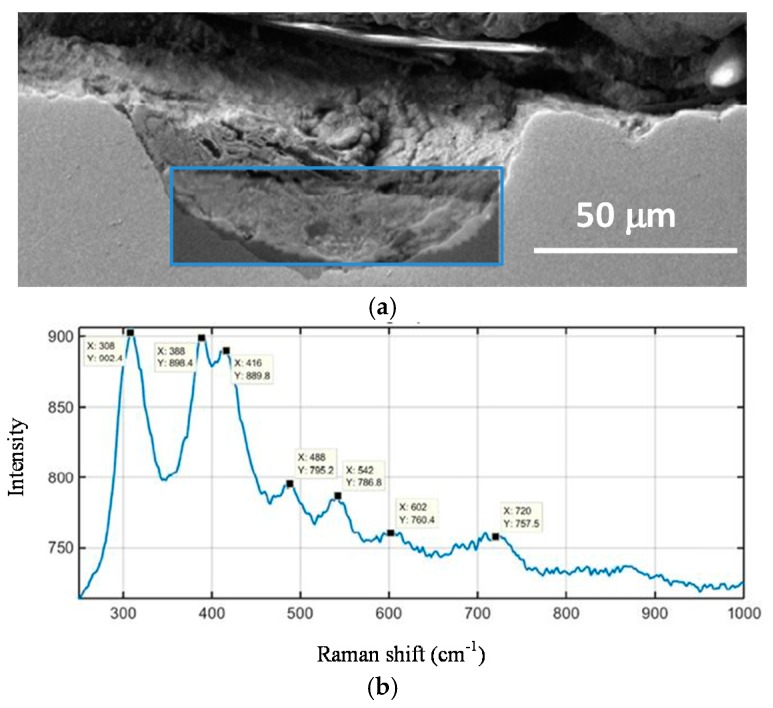
The rust present inside the pits formed on carbon steel substrates when exposed to severe marine atmospheres is almost entirely composed of akaganeite: (**a**) cross section affecting a deep pit; (**b**) Raman spectrum of rust inside the pit.

The thickness of the rust layer formed in marine atmospheres is not usually uniform, being thicker in some areas than in others, and the attack profile of the underlying steel generally shows the abundant formation of pits of variable depths [64]. Asami and Kikuchi [160] published an interesting study on in-depth distribution of rust components (determined by TEM/ED) on steel exposed to the atmosphere for 17 years under a bridge in a coastal-industrial region of Japan. They saw the aforementioned thick and thin areas within the rust layers and found that akaganeite was preferentially located in the thick areas and was scarce in the thin areas of the rust layers. The existence of akaganeite inside the pits formed on CS exposed for three months in a marine atmosphere with a high Cl^−^ deposition rate (1136 mg/m^2^·d) has been confirmed by RS in unpublished results obtained by the authors of this work (Figure 22).

In not highly aggressive marine atmospheres, consistent (consolidated), adherent, and continuous rust layers present a two-sublayer organisation, as has been seen above (Figure 19). However, the exposure of CS in very aggressive marine atmospheres can in certain circumstances lead to the formation of heterogeneous and anomalous thick rust layers. High times of wetness of the metallic surface and an atmosphere with a high Cl^−^ deposition rate lead to the formation of this type of rust. These thick rust layers tend to become detached from the steel substrate (exfoliated), leaving it uncovered and without protection and thus accelerating the metallic corrosion process [129]. The rust exfoliation phenomenon can only take place if such anomalous thick rust layers are formed, as has also been observed in studies carried out by other researchers [42,161]. In studies by the authors on CS corrosion in Cl^−^-rich atmospheres, the average Cl^−^ deposition rate needed to exceed a critical threshold of close to 300 mg/m^2^·d for exfoliation to take place; the annual steel corrosion at that atmospheric salinity was higher than 100 µm [129].

**Figure 23:**
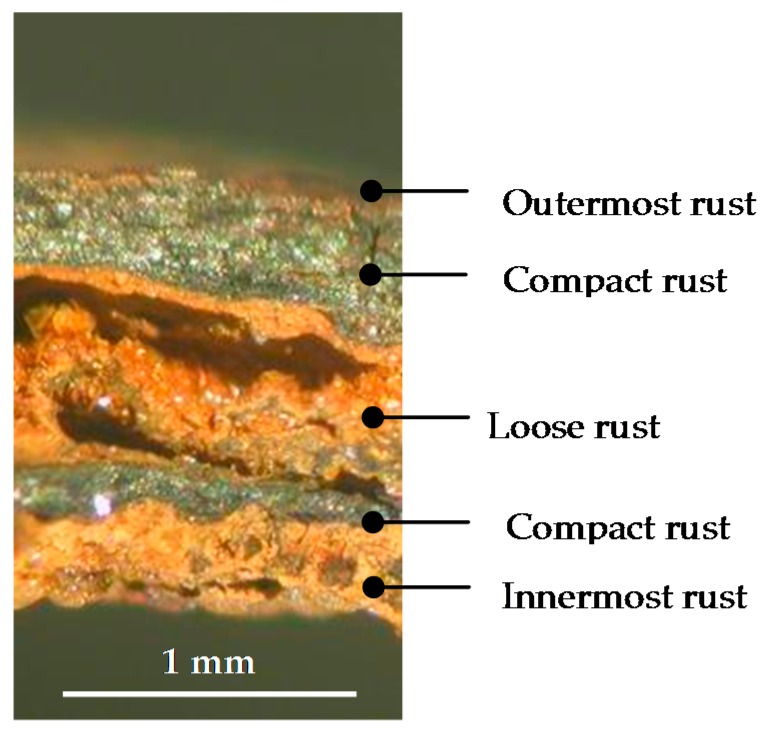
Optical micrograph of exfoliated (multilayered) rust formed on mild steel after one year in a very aggressive marine atmosphere (390 mg Cl^-^/m^2^·d) [129]. The characteristic of the different rust sublayer within the rust multilayer are described in Figure 24 [162].

**Figure 24:**
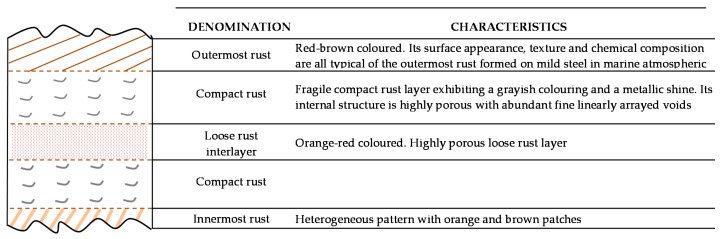
Schematic illustration of different rust sublayers in exfoliated rust (Figure 23). The denomination and characteristic of each rust layer is given [162].

The exfoliated rust layers are composed of multiple rust strata, this can clearly be seen in the cross section of Figure 23 and Figure 24. Observation by optical microscopy shows that in general the thick rust layer contains one or more strata of compact rust, exhibiting a greyish colouring and a metallic shine, whose number varied according to the area of the rust layer observed. With regard to the rust exfoliation mechanism, it is recommended to consult recent publications by the authors [129,153,162].

#### 6.2.5. Porosity

Voids of different sizes are formed among the rust particles in the corrosion layer, whose compactness depends on the rust particle size.

Important parameters for the protective ability of rust layers include their thickness, porosity and Specific Surface Area (SSA). According to Dillmann et al. [47] these parameters directly influence the amount of oxyhydroxides that will be in contact with the electrolyte and will be reduced. Thus, these characteristics of the rust layer directly influence the AC mechanisms.

The pore structure of the rust layer is clearly related with steel corrosion because various molecules and ions such as O_2_, H_2_O, and Cl^−^ diffuse through the rust layer in the corrosion process [146,163]. Despite this fact, few studies of rust pore structures have been reported. The pore size of atmospheric rust is in the range of up to 15 nm, and the highest peak always appears below 5 nm [151].

Dillmann et al. [47] characterised the overall porosity, pore diameter and SSA of pores in rust layers using different complementary methods: MIP, BET and SAXS. However, as the authors note, these methods do not provide quantitative data on the three-dimensional distribution of pores in the rust layer, their tortuosity or their connectivity; three other parameters about which information is desirable for AC modelling [41].

Ishikawa et al. [48,141,146] have published abundant information on this subject, using the adsorption method to obtain more complete information than the aforementioned techniques on a wide range of rust layers. The SSA of rust layers is evaluated by fitting the BET equation [164] to the adsorption isotherms of nitrogen (S_N_) and water molecules (S_W_) and using the cross-sectional area of nitrogen and water molecules (0.162 and 0.108 nm^2^, respectively) [165]. Among the results obtained using this technique, attention is drawn to the following which are considered particularly relevant:

(a) Estimation of rust particle size [141]

Ishikawa et al. apply adsorption of nitrogen molecules to estimate the rust particle size, successfully demonstrating that the SSA of the rust layer is a valid means of assessing its protective nature. They saw that the pores formed between larger particles were more accessible to O_2_, H_2_O and SO_4_^2−^ and Cl^−^ ions, which are important substances in the AC of steel.

**Figure 25:**
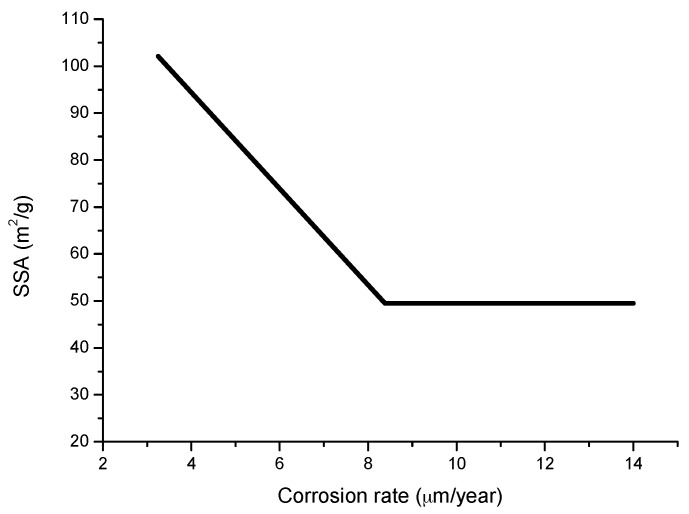
Trend plot of specific surface area (SSA) against corrosion rate for the rusts formed by exposing carbon steels at different bridges in Japan for 17 years [141].

**Figure 26:**
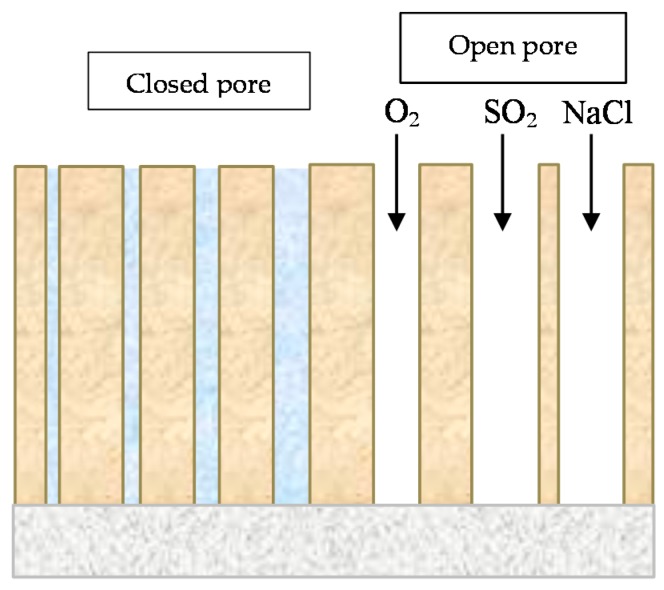
Schematic representation of pore filling by adsorption and capillary condensation of water, according to Ishikawa et al. [141].

Compact rust layers with a high S_N_ or a small particle size exhibit high corrosion resistance (Figure 25). The corrosion resistance of rust layers is a result of pore filling by the adsorption and capillary condensation of water [141], according to the proposed scheme shown in Figure 26.

(b) Pore size distribution of rusts formed on exposed steels [146]

**Figure 27:**
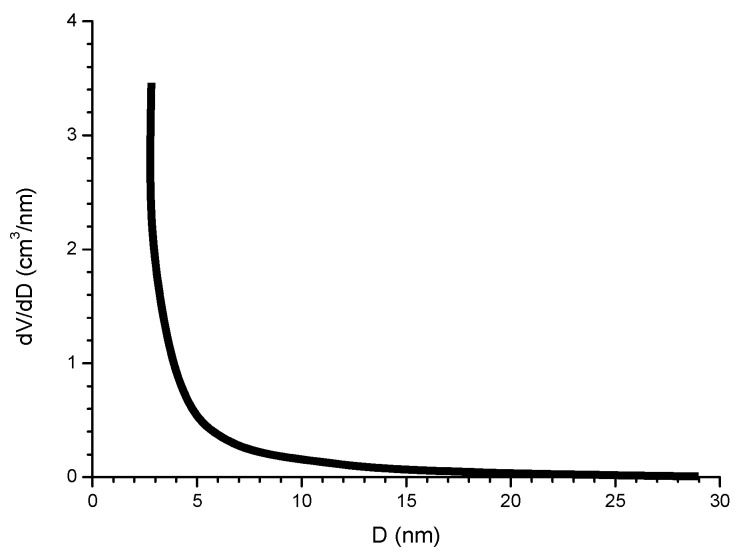
Trend plot of pore size distributions of the rust formed on steels exposed to coastal conditions [48]. V: adsorbed amount of N_2_; D: pore diameter.

Figure 27 depicts the pore size distribution trend curve of rusts formed on steels, as calculated by the Dollimore-Heal (D-H) method [165] from N_2_ adsorption isotherms. The curve rises steeply at pore diameters (D) of <~5 nm and still increases at D = ~2 nm, suggesting that these rusts contained micropores with a D < 2 nm.

(c) Influence of particle size and pore size distribution on rusts formed in marine atmospheres

Rusts formed in saline environments, such as marine or coastal regions or districts where deicing salts are used, show larger particle sizes than rusts formed in rural and urban areas, resulting in the formation of larger pores which act as voids between larger particles in the rust layer and facilitate further corrosion.

The Cl^−^ ion promotes the growth of rust particles to yield less compact rust layers composed of larger particles, which leads to a high corrosion rate of steels in the saline environment [48,146].

(i) Effect of chloride deposition rate on rust particle size [146]

**Figure 28:**
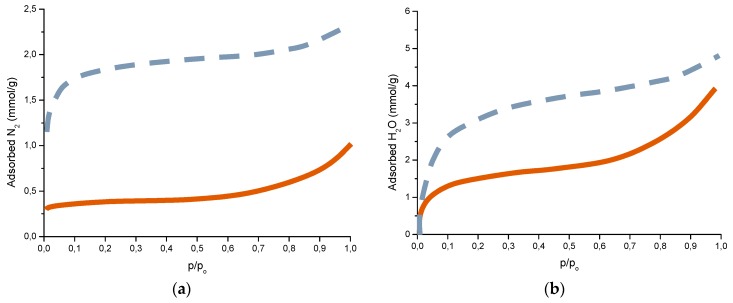
Adsorption isotherms of nitrogen (**a**) and water (**b**) on the rusts generated by exposing a carbon steel for 3 months at two atmospheres with different NaCl deposition rates [146]. 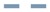 5.6 mg NaCl/m^2^; wet period: 56%; 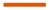 10.7 mg NaCl /m^2^; wet period: 82%; p/p_o_ is the relative pressure of water or relative humidity (RH).

Figure 28 shows the adsorption isotherms of nitrogen (S_N_) and water (S_W_) molecules on rusts obtained on CS exposed to atmospheres with different Cl^−^ deposition rates and times of wetness (TOW). S_N_ and S_W_ depend strongly on the Cl^−^ deposition rate and the TOW duration. Thus, the formation and growth of rust particles is influenced by both parameters. These results confirm that the development of rust particles in aqueous solutions and the particle growth of rusts is promoted by Cl^−^ ions.

(ii) Effect of chloride deposition rate on rust pore size [146]

The SSA (determined by N_2_ adsorption) (Figure 17) decreases as the NaCl content of the atmosphere increases (the micropores volume (MPV) shows a similar tendency), indicating that the rusts formed at coastal sites are agglomerates of large particles which give rise to large pores, consistent with the fast corrosion rate at the coast. This finding again suggests that NaCl promotes rust particle growth.

#### 6.2.6. Indices to Evaluate the Protective Capacity of Rust Layers

**Figure 29:**
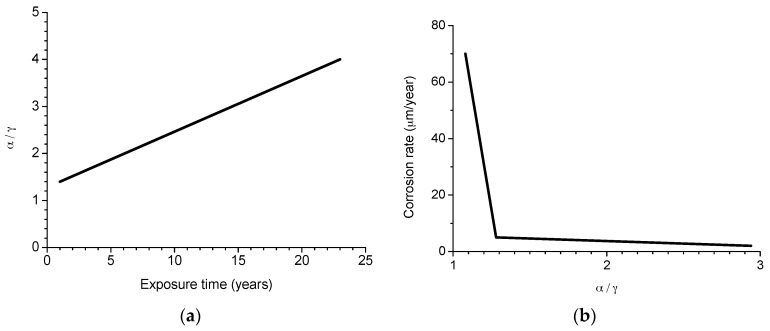
Relation between α/γ of the rust layer formed on weathering steel and exposure time (**a**) and corrosion rate (**b**). According to Yamashita and Misawa [166].

Yashamita and Misawa [166] found that the mass ratio of goethite to lepidocrocite contents (α/γ) in the rust layer formed on WS exposed in industrial and rural environments, determined by XRD, is a function of the exposure time, as is shown in Figure 29a. The α/γ ratio increases proportionally with exposure time due to the long-term phase transformation. The relationship between α/γ and the corrosion rate is shown in Figure 29b. It can be said that the corrosion rate decreases as the α/γ increases, and that α/γ > 2 is a necessary condition for the final protective rust layer. α/γ can be considered a useful index to evaluate the protective ability of the rust layer.

Kamimura et al. [167] and Hara et al. [142] later noted that this tendency for the α/γ mass ratio to increase with exposure time was not seen in marine atmospheres where the corrosion species akaganeite (β) and magnetite (M) also form on steel. In contrast, they saw that the relationship between goethite and the total mass of lepidocrocite, akaganeite and magnetite (α/γ*) was related to the corrosion rate, even in coastal environments with more than 0.2 mg/dm^2^·d of airborne sea salt particles. When the α/γ* ratio exceeded a certain critical threshold, steel corrosion was less than 10 µm/year.

Despite the fact that magnetite is a conducting phase, Dillmann et al. [47] consider it to be protective because of its relatively good stability, suggesting a new protective ability index (α*/γ*):

α*/γ* = (α + M)/(γ + β),(20)

## 7. Mechanisms of Steel in MAC

### 7.1. First Researches

As has been noted in the introduction to this review, it is rather surprising that despite the great practical importance of this issue it has only recently that it has started to attract the interest of corrosion scientists.

It is well known that the presence of atmospheric pollutants (natural or anthropogenic) notably accelerates the AC process of CS. The two most common pollutants, which have drawn the majority of research efforts, are SO_2_ and marine chlorides. In principle most of the attention has been focused on SO_2_, and considerable progress has been made in this respect [4]. However, as Nishimura et al. [10,39] point out, it was not until the final decade of the 20th century that major research started to be carried out on the fundamental mechanisms of rust formation in Cl^−^-rich marine atmospheres. Until then very little research was undertaken in this field, the most notable being the work of Keller [103], Feitknecht [24], Henriksen [168] and Misawa [169,170,171].

Keller in 1948 [103] reported three basic chlorides obtained by partial precipitation from FeCl_2_ solution in various concentrations, noting that these three basic chlorides were presumably the precursors of GR1. Feitknecht [24] reported the existence of chloride accumulations (nests) containing FeCl_2_ in the rust layers formed on steel exposed in coastal areas and their role in stimulating AC. Henriksen [168], using autoradiography, noted that the AC of CS in marine atmospheres starts at weak spots in the oxide film. Na^+^ and Cl^−^ in the aqueous adlayer migrate to these weak spots and once Cl^−^ has been adsorbed and corrosion has begun, Na^+^ migrates to the cathodic areas. Misawa et al. [169,170,171], who had carried out important basic research on the formation mechanisms of the different AC products of iron, noted in their work the formation of an intermediate compound, GR1, in marine atmospheres, also in accordance with Keller [103].

At around the same time, Barton [4], in his important book on AC, pointed out three causes which explained the high corrosion rates of steel exposed to marine atmospheres: (a) the increase in the ionic conductivity of the aqueous adlayer due to the presence of ionising substances (chlorides); (b) the hygroscopic nature of the Cl^−^-containing corrosion products formed; and (c) the solubility of the latter, unlike the stable basic chlorides that form in the case of other metals (Cu, Zn, etc.), indicating that the mechanism which governs the effects of Cl^−^ ions in AC had not been completely explained.

In addition to the above, two other causes of enormous importance should be mentioned: (a) the strong cathodic depolarising role of Cl^−^ ions, accelerating the cathodic process by tens and hundreds of times, as formulated in 1961 by Rozenfeld [18]; and (b) the catalytic role of chlorides [23,172]. With regard to the latter effect, it is noted that the anodic reaction generates cations by dissolution and H^+^ by hydrolysis of the dissolved cations. Both Fe^2+^ and H^+^ require neutralisation, which is accomplished by the ingress of chlorides. The locally higher Cl^−^ concentration enhances local metal dissolution, then draws more Cl^−^ and enhances dissolution even further [23]. Migration of Cl^−^ to the corroding substrate is facilitated by its high permeability in the rust layer. As noted in [23], this is a feedback mechanism, sometimes referred to as autocatalytic. The high concentrations of Cl^−^ in the inner rust layer will facilitate the formation of akaganeite, as was also previously noted by Keller [103] and Misawa [169].

According to Misawa et al. [169], when the aqueous adlayer on the metal surface is neutral or slightly acidic, Fe(OH)_2_ cannot be formed, but various Fe(II) hydroxo-complexes may be formed, depending on the existing anion in the aqueous solution. Fe(II) hydroxo-complexes thus formed are oxidised by dissolved oxygen, resulting in lepidocrocite through an intermediate GR.

Fe(II) hydroxo-complexes→GR→γ-FeOOH,(21)

In marine atmospheres, the intermediate generally has a GR1 structure that forms in the presence of Cl^−^ ion [96,107,108]. GR1 is converted to black magnetite by slow oxidation in solution and this reaction is considered to correspond to the formation of magnetite in the underlying rust layer where the oxygen supply is limited. Also, akaganeite can be obtained by the dry oxidation of solid β-Fe_2_(OH)_3_Cl precipitated from the slightly acidic solution in the presence of Cl^−^ ions.

Fe(II) hydroxo-complexes→GR1→β-Fe_2_(OH)_3_Cl→β-FeOOH, (22)

Worch et al. reported in 1983 [173] that GR1 is often seen on iron and steel exposed to marine environments and that chloride may also be involved catalytically in the formation of akaganeite. Akaganeite is produced only in the presence of sufficient concentrations of Cl^−^ [107,108].

Askey et al. [25] suggest a cyclical rust formation process, similar to the acid regeneration cycle proposed by Schikorr [26,174] for the action of SO_2_ in iron, by which the accumulation of Cl^−^ ions in the underlying steel gives rise to the formation of FeCl_2_, which hydrolyses water according to

2FeCl_2_ + 3H_2_O + ½O_2_→2FeO(OH) + 4HCl, (10)

Releasing HCl. It should be noted that this represents a reaction cycle in which rereleased HCl will react with iron to form fresh FeCl_2_. Once started, therefore, the cycle will be independent of incoming HCl. Corrosion will continue until the Cl^−^ ions are removed (possibly by the washing away of FeCl_2_).

### 7.2. The Fundamental Role of Akaganeite in the Atmospheric Corrosion Process of Steel in Marine Atmospheres

**Figure 30:**
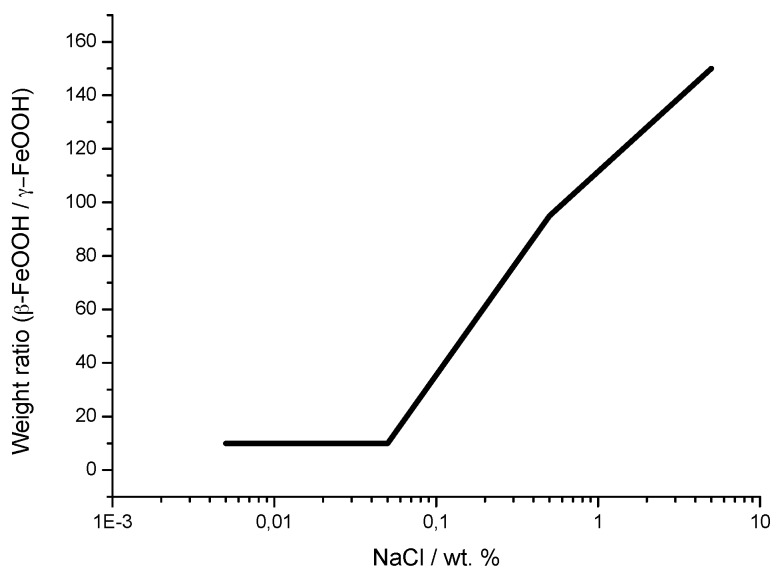
Weight ratio of β-FeOOH to γ-FeOOH as a function of NaCl concentration [39].

A fundamental advance in relation with the role played by akaganeite in the AC process of steel in marine atmospheres was made by Nishimura et al. in studies carried out in the last decade of the 20th century [10,39]. Nishimura et al. in 1995 used a wet and dry corrosion test to study the relationship between steel corrosion resistance and NaCl concentration, analysing the corrosion products by in-situ XRD [39]. The steel corrosion rate increased as the NaCl concentration rose, and a very strong increase in the akaganeite/lepidocrocite weight ratio was observed from a NaCl concentration of 0.05 wt % (Figure 30).

**Figure 31:**
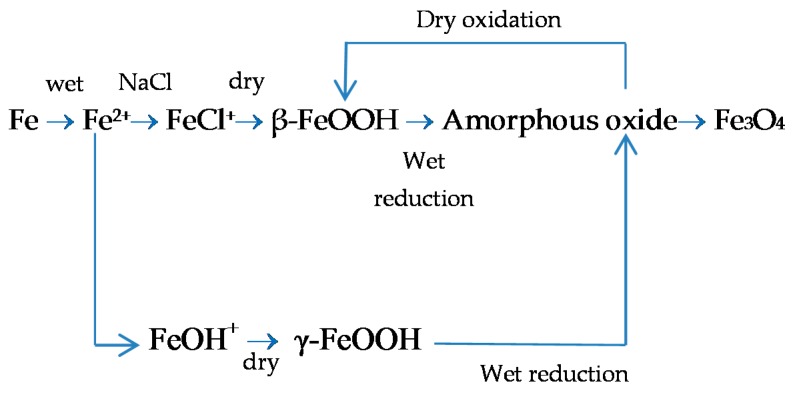
Rusting model of iron in wet and dry corrosion condition containing NaCl [39].

Akaganeite was reduced to an amorphous intermediate oxide during the wet stage of the cycle and reproduced in the dry stage, giving rise to the proposal of the following rusting model of iron in wet and dry corrosion in the presence of NaCl (Figure 31).

Later, in the year 2000, continuing with the in-situ XRD technique but here in combination with alternating current impedance, Nishimura et al. observed the transition of akaganeite from GR1 in the dry process; the amount of GR1 also depended on the Cl^−^ ion concentration [10]. After dripping a Cl^−^ solution (3% Cl^−^) on the steel surface, the dry process progressed with the formation of akaganeite at a high corrosion rate. When a low Cl^−^ concentration was used (0%–0.3% Cl^−^), lepidocrocite was formed from Fe(OH)_2_ instead of akaganeite and the corrosion rate was low.

**Figure 32:**
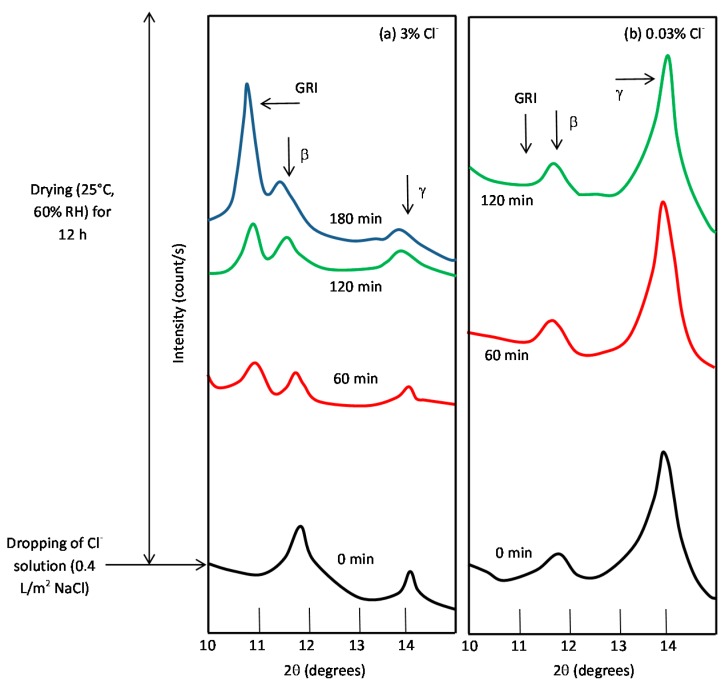
In-situ X-ray diffraction (XRD) results in wet and dry corrosion test using (**a**) 3% Cl^−^ and (**b**) 0.03% Cl^−^ solution [10].

The integral intensity of akaganeite decreased after dripping a Cl^−^ solution, which implied that akaganeite was consumed in the wet process. After 60 min in the dry process of the cycle the presence of GR1 was detected. GR1 could still be detected after 180 min (Figure 32), but disappeared after 12 h of testing, indicating that the transformation to akaganeite was complete.

Quantitative analysis of the identified phases was carried out using an XRD standard method. Akaganeite was the most abundant crystalline phase in the iron rust, and its proportion grew considerably as the Cl^−^ concentration increased, as was previously seen [39]. The other large phase was goethite. In contrast, the amounts of lepidocrocite and magnetite were low. These two phases were not affected by the Cl^−^ concentration. Nishimura et al. concluded that in a Cl^−^-rich environment the corrosion process was dominated by the formation of akaganeite, rather than lepidocrocite, which acts as the oxidation agent that accelerates corrosion in Cl^−^-free environments [38].

The authors carried out XPS and TEM observations on those portions of iron rust that could not be detected by XRD. It was determined that they contained large amounts of spinel oxide (magnetite structure) with bivalent/trivalent iron. This spinel oxide may have been formed by reduction of akaganeite during the wet process of the cycle.

These findings mark a turning point in the knowledge of the MAC mechanisms of steel, determining that steel corrosion progresses by the formation of akaganeite from GR1 (dry stage or “drying-out stage” following the terminology of Stratmann [38]) and its reduction (wet stage).

### 7.3. Initial Stages of MAC

In marine atmospheres, corrosion is generally driven by the deposition of hygroscopic sea salt aerosols that absorb moisture from the environment and form salt droplets. These aerosols range from a few angstroms to several hundred microns in diameter. Lan et al. [175] point out that coarse sea salt particles are those that contribute most to CS corrosion in marine atmospheres. In coastal marine locations (<2 km from the shoreline) the most common aerosols deposited are in the “coarse mode” size range: 1–100 μm in diameter [57].

Li and Hihara [55] studied salt particle deposition and the initial stage of MAC at severe marine test sites. They found both: (a) small sea-salt particles (D < 5 μm) and sea-salt clusters (D < 10 μm) formed by dehydration on the steel substrate that did not corrode under relatively small seawater droplets (D < 30 μm); and (b) sea-salt clusters integrated with iron corrosion products formed on the steel substrate that did corrode from larger seawater droplets (D > 30 μm). The corrosion that occurred under larger seawater droplets (D > 30 μm) showed the typical characteristics of droplet corrosion, with Cl^−^ and Na^+^ ions migrated to the central anode and peripheral cathode, respectively. The corrosion products were identified as lepidocrocite.

These same researchers [176,177], in a laboratory study in which they manually deposited NaCl droplets of different diameters on CS steel, used RS to analyse the very initial stage of NaCl particle-induced corrosion. They found that corrosion did not initiate under NaCl droplets with diameters of less than 45 μm after 6 h (at 80% RH). At larger NaCl droplet diameters, corrosion initiated quickly under the droplets in the form of pitting. In-situ and ex-situ Raman spectra show the formation of GR in regions close to the anodic sites and the precipitation of lepidocrocite clusters over cathodic sites surrounding the GR region. Magnetite was detected mostly in the rust clusters formed in the transitional region from GR to lepidocrocite. Upon exposure to ambient air, GR transformed to the more stable lepidocrocite due to oxidation. Li and Hihara underline the need for more research effort in droplet electrochemistry [178].

Risteen et al. [179] recently used a new methodology to study corrosion under NaCl solution droplets ranging in diameter from 20 to 1000 μm. They also observed the dependence of the occurrence of corrosion on drop size, noting that this behaviour appears to be strongly dependent on the microstructure and surface finish: corrosion initiation on 1010 steel was dominated by manganese sulfide inclusions when a mirror surface finish was maintained. In contrast, for high purity iron, initiation was dominated by surface roughness.

Ohtsuka and Tanaka have recently used RS to carry out a study of changes in rust composition on CS over six days of cyclic exposure: 4 h wet (90% RH)/4 h dry (10% RH) in the presence of NaCl droplets (0.93 and 0.11 mg/cm^2^) [180]. The NaCl solution was first dripped and immediately dried in a vacuum desiccator. A Raman spectrum was recorded every 15 min.

**Figure 33:**
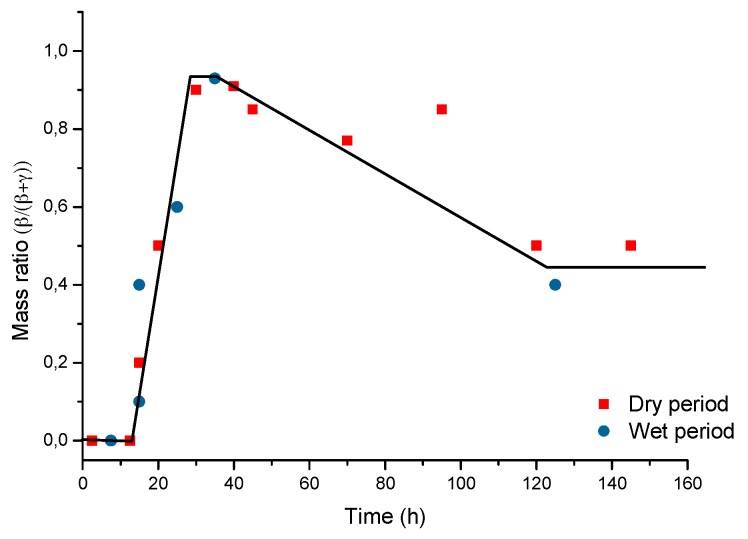
Change of mass ratio of (β-FeOOH /(β-FeOOH + γ-FeOOH)) of rust on weathering steel during the exposure of dry and wet periods in the presence of NaCl deposition at 0.93 mg/cm^2^ [180].

The Raman spectra of the rust surface in the presence of 0.93 mg/cm^2^ NaCl deposits corresponded to lepidocrocite and magnetite in the initial 12 h of exposure. After 12 h of exposure, akaganeite started to form, and its molar ratio on the rust surface increased to 90% at 30 h of exposure (Figure 33). They assume that in order for akaganeite to form, a lepidocrocite + magnetite rust layer of some thickness is required. The Raman spectra further changed after 30 h of exposure, when lepidocrocite again emerged. The reappearance of lepidocrocite is assumed to be caused by the capture of Cl^−^ ions in the akaganeite, resulting in a decrease in the free Cl^−^ ions in the aqueous adlayer.

When the amount of NaCl deposit is decreased to 0.11 mg/cm^2^, the steel surface does not reach high enough concentrations to form akaganeite. Only after repeated wet/dry cycles may a spot with a high concentration of NaCl emerge on the surface and akaganeite form on that spot.

### 7.4. Formation and Growth of the Corrosion Layer

Once the corrosion process has started on the steel surface it will be necessary to consider the possible mechanisms that take place in the formation and growth of the rust layer, where, as is known, the corrosion products transform from one compound to a more stable form and may involve any of a number of processes including hydrolysis, nucleation, crystallisation, precipitation, dehydration, thermal transformation, dehydroxylation, etc. [91]. Temperature, time and pH are the main factors governing such transformations [97].

In 1965, Evans [181] formulated the first electrochemical method for atmospheric rusting, in which the oxidation of iron (wet periods)

Fe→Fe^2+^ + 2e^−^, (1)

Is balanced by the reduction of ferric rust to magnetite

Fe^2+^ + 2 FeOOH→Fe_3_O_4_ + 2H^+^, (23)

Later, after partial drying of the pore structure of the rust (dry period), magnetite is reoxidised by oxygen that now has free access through the pores due to gas diffusion

Fe_3_O_4_ + 3/2 O_2_ + H_2_O→3 γ-FeOOH, (24)

The autocatalytic cycle responsible for the fact that rust promotes further rusting involves alternate reduction and reoxidation of the preexisting rust.

Subsequently, Stratmann et al. [182], in an electrochemical study of phase transitions in rust layers, experimentally showed that the oxidation of magnetite to lepidocrocite, as proposed by Evans, was not possible. Stratmann et al. used a combination of magnetic and volumetric measurements to show that when a prerusted iron sample is wetted, iron dissolution is not immediately balanced by a reaction with oxygen, but rather by reduction of the preexisting rust

γ-FeOOH (lepidocrocite) + H^+^ + e^−^→γ-Fe.OH.OH (reduced lepidocrocite), (25)

With later reoxidation of the reduced species

2 γ-Fe.OH.OH + 1/2 O_2_→2 γ-FeOOH + H_2_O, (26)

Thus, Stratmann [38] proposed dividing the AC mechanism of pure iron into the following three stages: wetting of the dry surface, wet surface, and drying-out of the surface (see Figure 2).

**Figure 34:**
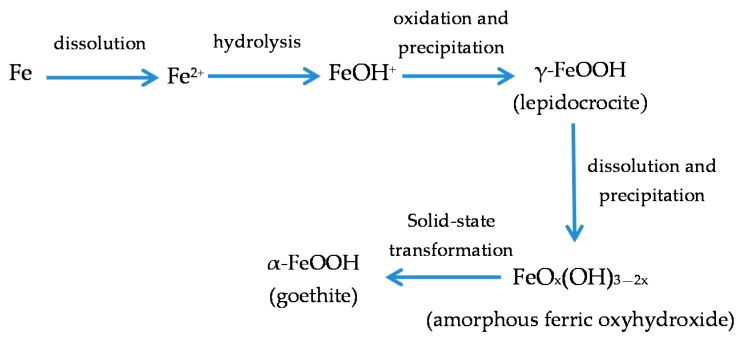
Mechanism for the rusting process according to Misawa [170].

Misawa [170] notes the following mechanism for the rusting process (Figure 34):

(a) In the first stage of rusting the aerial oxidation of ferrous ions, dissolved from the steel into a slightly acidic thin water layer formed by rain on the steel surface, leads to the precipitation of lepidocrocite. Fine weather accelerates the precipitation and crystallisation of lepidocrocite by drying.
(b) The lepidocrocite is formed on the steel surface and transformed to amorphous ferric oxyhydroxide and goethite during the atmospheric rusting process. The amorphous ferric oxyhydroxide transforms to goethite by deprotonation using hydroxyl ions provided by the rainwater.

Another important aspect to consider is how the rust layer grows. Horton [183] in 1964 observed that rust layers grow by several mechanisms: (i) by iron ions diffusing outward through the rust to form fresh rust at the air-rust interface; (ii) at the steel-rust surface; and (iii) within the rust layer to fill pores and cracks. It was the first time that this observation was reported in scientific literature. Years later, Burger et al. [184] using an ingenious technique known as the “gold marker method”, addressed the following two aspects:

(a) the location at which precipitation of corrosion products occurs within the corrosion system (steel/rust/atmosphere), and
(b) the structural evolution of the corrosion product layer during wet/dry cycles.

With regard to (a), they observed a significant contribution of inward diffusion of oxidant through the corrosion product layer. With regard to (b) they note that the continuous decrease in the reactivity of the corrosion product layer seems to be related with a two-step process in the corrosion mechanisms: the preliminary formation of ferrihydrite (a highly reactive hydrated iron oxide) close to the metal/rust interface, followed by its progressive transformation into goethite, a more stable oxyhydroxide. This progressive transformation may be the consequence of incremented cyclic reduction/reoxidation reactions which are not completely reversible. As these cyclic electrochemical reactions require electrical contact between the reactive phase and the metallic substrate, and given the complex morphology of the corrosion patterns, an important outlook is to take into account the connectivity and conductivity of the different phases constituting the corrosion product layer and their influence on its structural evolution. Due to the expansive nature of the corrosion products, mechanical stresses may develop in these materials, thus inducing two opposing effects: pore blocking and formation of cracks/spalling in the rust layer.

In the last decade great advances have been made in the understanding of AC mechanisms. As has been mentioned above, many of these advances have been due to the French research groups of Professors Legrand and Dillmann [40,41,44,47,93,131,185,186]. Both groups have made important advances in: (a) the electrochemical reactivity of the ferric phases that constitute rust; (b) the localisation of oxygen reduction sites; (c) the decoupling of anodic and cathodic reactions; (d) in-situ characterisation of reduction and reoxidation processes, etc.

With a view to the development of a model of the AC process that can predict the long term AC behaviour of iron, they note the need to consider several important parameters in order to describe rust layer: average lepidocrocite fraction, thickness, average porosity, tortuosity and specific area, connectivity of the phases inside the rust layer, etc. [41,47]. As these researchers note, there is still a long way to go before long-term AC mechanisms are fully clarified.

As has been mentioned several times in this paper, considerable advances have been made in the knowledge of AC mechanisms in atmospheres polluted with SO_2_ (e.g., urban and industrial atmospheres) while less progress has been made on corrosion mechanisms in marine atmospheres. In addition to the proposals of Nishimura et al. [10,39] referred to in 7.2., other authors have made contributions relating to the subject of MAC which will be enumerated below, and more are sure to appear in the forthcoming years, considering the growing interest of researchers in this field of knowledge.

In 1988 Nomura et al. [187] applied conversion electron MS to study the formation of akaganeite on iron in a NaCl (3 wt %) solution. On the basis of the study on early stages of Fe(OH)_2_ formation, the reaction that takes place on the iron surface in a Cl^−^ solution can be expressed as follows:

O_2_ + 2H_2_O + 4e^−^→4OH^−^, (2)

Fe→Fe(OH)^+^→Fe(OH)_2_^+^, (27)

In conditions of high dissolved oxygen (initial stages)

Fe(OH)_2_^+^ + 2OH^−^→Fe^3+^(OH)_4_^−^, (28)

2Fe(OH)_4_^−^→2FeOOH + 2H_2_O + 2OH^−^, (29)

Poorly crystalline FeOOH is considered to deposit on the iron surface by the initial corrosion reaction listed above.

However, in conditions of low dissolved oxygen, when a first rust layer is formed, the supply of OH^−^ (Equation (28)) is suppressed, and Cl^−^ ions begin to have a relatively strong affinity to the iron(III) ion, thus iron(III) oxyhydroxide complexes containing Cl^−^ may be formed.

Fe(OH)_2_^+^ + (2 − X)OH^−^ + XCl^−^→Fe[OH_4−X_Cl_X_]^−^, (30)

Before the start of the polymerisation process to form rust according to the following equation:

2Fe[OH_4−X_Cl_X_]^−^ (polymerisation)→2β-FeOOH (Cl_X_^−^) + H_2_O + 2_(1−X)_OH^−^, (31)

In short, when unstable oxyhydroxide is first formed on iron, lepidocrocite is formed on the surface where dissolved oxygen has easy access, and then akaganeite and magnetite start to be produced by the transformation of the Fe(OH)_2_ complex containing Cl^−^ at the intermediate surface between the lepidocrocite layer and the iron substrate and by the slow oxidation of iron, respectively, because the supply of dissolved oxygen to the intermediate layers is restricted by the top lepidocrocite layer.

It is relevant to note at this point the important laboratory studies carried out by Refait and Genin [113,115] and subsequently by Remazeilles and Refait [112,114] on the formation conditions of Fe(II) hydroxychlorides, GR1 and akaganeite previously mentioned in Section 5.

More recently, Ma et al. [188,189], using XRD and IRS, detected the formation of akaganeite in the inner rust layer accompanied by an acceleration of the corrosion rate on steel exposed to very severe marine atmospheres. After six months of exposure the akaganeite content and the corrosion rate decrease and akaganeite is gradually transformed into maghemite until it completely disappears. The authors speculate on the need to exceed a critical chloride threshold in order for akaganeite to form.

**Figure 35:**
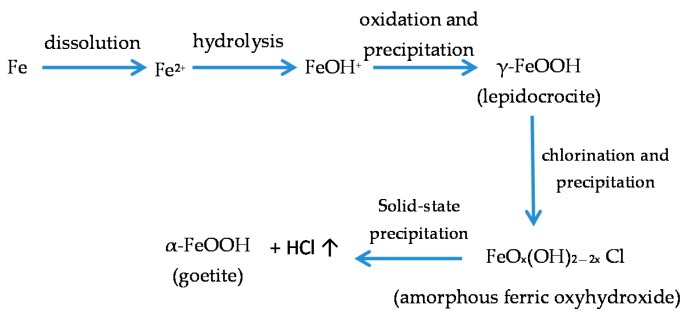
In marine atmospheres with low chloride deposition rates, Cl^−^ content facilitates the transformation of lepidocrocite into goethite [188,189].

In atmospheres with less Cl^−^ pollution akaganeite was not formed, though the Cl^−^ content facilitated the transformation of lepidocrocite into goethite (Figure 35). The wet/dry cycle accelerates these transformation processes, and especially in the dry cycle HCl is released into the environment.

### 7.5. Proposal of an Overall Mechanism for the MAC Process of Steel

The composition of the rust layer depends on the conditions in the aqueous adlayer and thus varies according to the type of atmosphere. It is unanimously accepted that lepidocrocite is the primary crystalline corrosion product formed in the atmosphere. As the exposure time increases and the rust layer becomes thicker, the active lepidocrocite is partially transformed into goethite and magnetite.

In mildly acidic solutions lepidocrocite is transformed into goethite. Schwertmann and Taylor established that the transformation occurs in solution through different steps: dissolution of lepidocrocite, formation of goethite nuclei, and nuclei growth [190].

Magnetite may be formed by oxidation of Fe(OH)_2_ or intermediate ferrous-ferric species such as GR [119], but also by lepidocrocite reduction in the presence of a limited oxygen supply [119,120]:

2γ-FeOOH + Fe^2+^→Fe_3_O_4_ + 2H^+^, (17)

Thus it is not surprising that magnetite is usually detected in the inner part of rust adhering to the steel surface, where oxygen depletion may occur.

With a broader view, Ishikawa et al. [121] and Tanaka et al. [122] found that the formation of magnetite particles was caused by the reaction of dissolved ferric species of oxyhydroxides with ferrous species in the solution. The formation of magnetite rust can be represented by the following cathodic reaction:

Fe^2+^ + 8FeOOH + 2e^−^→3Fe_3_O_4_ + 4H_2_O, (18)

In marine atmospheres, where the surface electrolyte contains chlorides, akaganeite is also formed. How does akaganeite form? The high Cl^−^ concentration in the aqueous adlayer on the steel surface gives rise to the formation of FeCl_2_, which hydrolyses the water [25]:

2FeCl_2_ + 3H_2_O + ½O_2_→2FeOOH + 4HCl, (10)

Notably raising the acidity of the electrolyte. At the steel/corrosion products interface, where Cl^−^ ions can accumulate, large Cl^−^ concentrations and acidic conditions give rise to akaganeite formation after the precipitation of ferrous hydroxychloride (β-Fe_2_(OH)_3_Cl), a very slow process requiring the transformation of metastable precursors [112,113].

As Remazeilles and Refait point out, large amounts of dissolved Fe(II) species and high Cl^−^ concentrations are both necessary for akaganeite formation [114]. The oxidation process of ferrous hydroxychloride which leads to akaganeite formation passes through different steps via the formation of GR1 intermediate compounds. The whole oxidation process can be summarised as follows [99,112,113,114,115]:

FeCl_2_→β-Fe_2_(OH)_3_Cl→GR1→β-FeOOH, (16)

Thus requiring a relatively long time. This time will depend on the environmental conditions: temperature, Fe^2+^, Cl^−^ and OH^−^ conditions, O_2_ flow, etc. The acid environment at the steel/rust interface also leads to an acceleration of corrosion of the underlying steel and pitting [23,191], where the dominant cathodic reaction is hydrogen evolution.

2H^+^ + 2e^−^→H_2_, (3)

**Figure 36:**
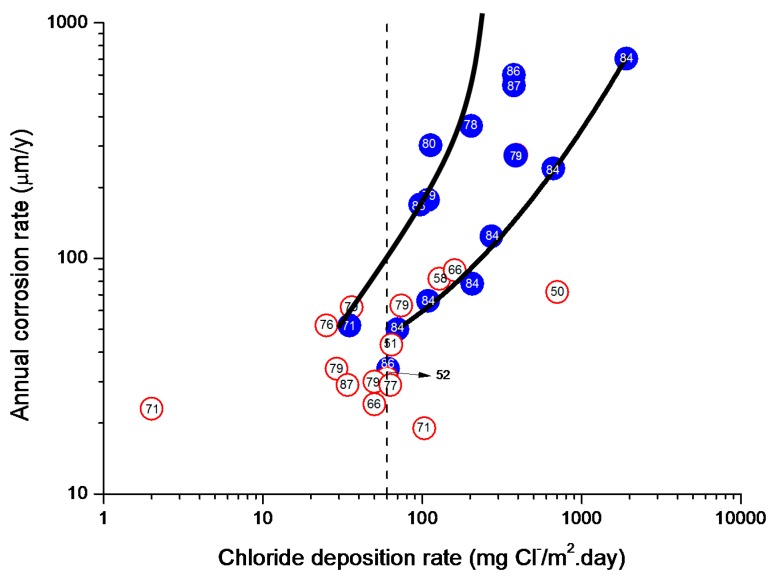
Corrosion rate of mild steel during the first year of atmospheric exposure as a function of the annual average chloride deposition rate at the exposure site. The points of the graph, represented by circles, include an indication of the annual average RH at exposure site. Blue circle represent test site where akaganeite had been identified, and white circle represent test sites where it had not been possible to identify it by XRD [192].

There has been much speculation about the need to exceed a critical atmospheric salinity threshold for akaganeite formation to take place. Morcillo et al. provide a general indication of the environmental conditions in the atmosphere which lead to akaganeite formation: annual average RH around 80% or higher and simultaneously an annual average Cl^−^ deposition rate of around 60 mg/m^2^·d or higher [192] (Figure 36). This confirms the laboratory experiments carried out by Remazeilles and Refait [114], which indicate that a high Cl^−^ concentration is not the only condition for akaganeite formation. The medium must also be characterised by large dissolved Fe^2+^ concentrations, as occur during the high TOW of the metallic surface in high RH atmospheres.

**Figure 37:**
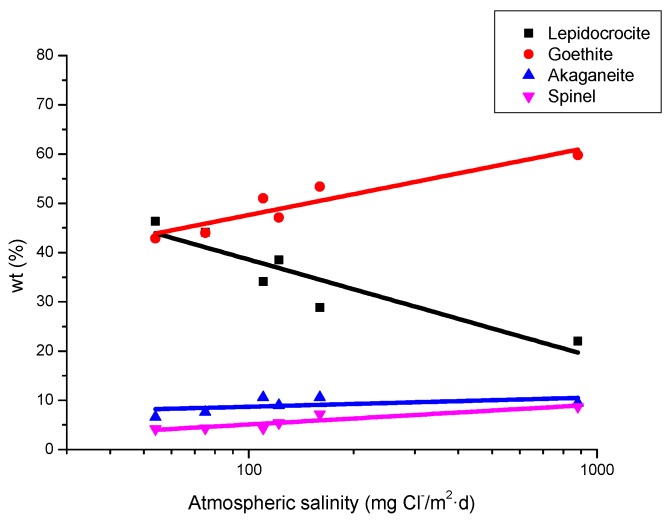
Variation of rust phases content in rusts formed on mild steel exposed during 3 months in marine atmospheres with different levels of salinity [95].

With regard to the corrosion products that form on CS in this type of atmospheres, Table 4 has been prepared using field data obtained by the authors in different studies carried out at various sites in Spain [64,150,193] and allows the following facts to be deduced: (a) in marine atmospheres with extremely low Cl^−^ deposition rates (Ponte do Porto) only lepidocrocite and goethite phases form, the latter in a practically insignificant proportion; (b) above a certain atmospheric salinity, akaganeite and spinel (magnetite/maghemite) phases appear; (c) the akaganeite and spinel contents increase notably as the atmospheric salinity rises (Cabo Vilano-2 and Cabo Vilano-3); and (d) it can clearly be seen how an increase in the Cl^−^ deposition rate is accompanied by a drop in the lepidocrocite phase content of the rust and a rise in the goethite, akaganeite and spinel contents. This fact is well seen in Figure 37, which shows rusts formed after 3 months exposure of CS in marine atmospheres with different salinities [95].

Akaganeite could be reduced electrochemically in the corrosion process, being consumed in the wetting of the metallic surface [10]. Lair et al. [40] experimentally saw the high reducing capacity of akaganeite in comparison with other oxyhydroxides, reporting the following order: akaganeite > lepidocrocite >> goethite.

This explains the high magnetite contents found in the rust formed on steel exposed to atmospheres with heavy Cl^−^ ion pollution. As Hiller noted some time ago, the rust formed in atmospheres with high Cl^−^ deposition rates contains more magnetite than that formed in Cl^−^ free atmospheres [123].

Rust layers present considerable porosity and cracking. Ishikawa et al., examining the textures of rusts, note that the rusts formed at coastal sites are agglomerates of large particles and have larger pores than rusts formed at rural and urban sites. NaCl promotes rust particle growth, resulting in the formation of larger pores and voids between larger particles in the rust layer and facilitating further corrosion [145,163]. The SSA of rusts decreases as salinity increases, enlarging the diameter of the pores and forming less and less compact rust layers with low protective properties.

Thus the compactness of the corrosion product layers formed is dependent on the salinity of the atmosphere at the exposure site. At low Cl^−^ deposition rates, even when the time of wetness (TOW) of the metallic surface is high, relatively consistent (less porous) layers, whose thickness does not usually exceed 100 μm, are formed. However, high Cl^−^ deposition rates lead to the formation of very porous rust layers showing cracks and even flaking and exfoliation [129].

**Figure 38:**
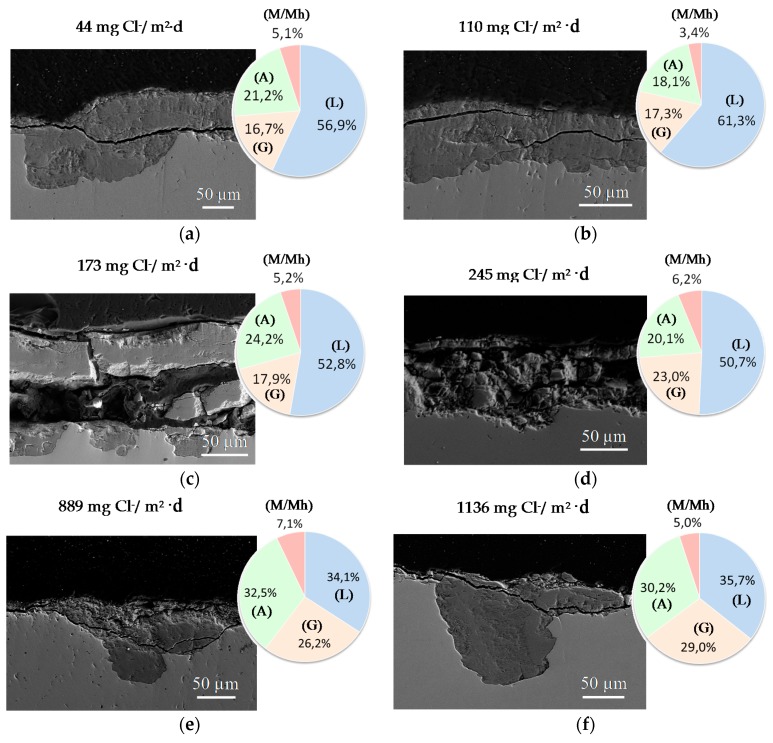
Variation in rust layer structure with atmospheric salinity: 44 mg Cl^−^/m^2^·d (**a**); 110 mg Cl^−^/m^2^·d (**b**); 173 mg Cl^−^/m^2^·d (**c**); 245 mg Cl^−^/m^2^·d (**d**); 889 mg Cl^−^/m^2^·d (**e**); 1136 mg Cl^−^/m^2^·d (**f**). Carbon steel specimens were exposed for three months in different marine atmospheres. The circles indicate the content of different phases in the rust, information obtained by XRD (RIR) of powdered rust [64]. L: lepidocrocite; G: goethite; A: akaganeite; M: magnetite and Mh: maghemite.

In Figure 38 it is possible to see the variation in the structure of the rust layer as the atmospheric salinity rises. While at relatively low atmospheric salinities (44 and 110 mg Cl^−^/m^2^·d) the rust layers are fairly compact (though they can show the presence of longitudinal cracks), at higher atmospheric salinities (173 and 245 mg Cl^−^/m^2^·d) the rust layers present abundant cracking which facilitates their subsequent detachment (exfoliation), as can clearly be seen at the highest atmospheric salinities (889 and 1136 mg Cl^−^/m^2^·d) [64].

Figure 38 also indicates how the content of the different phases in the rust layers varies as the atmospheric salinity rises. The lepidocrocite phase decreases while the goethite and akaganeite phase contents increase [64].

**Figure 39:**
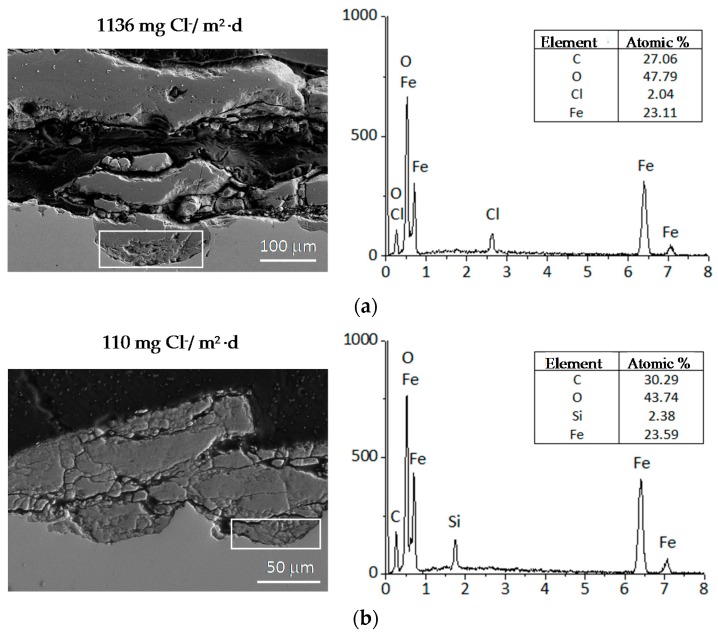
Formation of pits in steel substrate exposed to two atmospheres of different salinities. The EDS signal for Cl is more intense in the atmosphere of higher salinity [64].

The base steel shows the formation of pits when exposed to marine atmospheres. As the atmospheric salinity rises, pitting becomes more significant and the Cl signal obtained by EDS inside the pits also rises (Figure 39) [64]. As has been seen in Section 6 (Figure 22), there is a strong presence of akaganeite in the interior of the pits formed on the base steel in severe marine environments.

Thus there seem to be two notably different situations with regard to the mechanisms involved in the MAC of CS: (a) establishment of a consistent (consolidated), adherent and continuous rust layer (at low Cl^−^ deposition rates); and (b) formation of a thick rust layer that is easily detached (exfoliated) from the base steel, leaving large areas uncovered (at high Cl^−^ deposition rates) [193].

When a consolidated layer of corrosion products remains on the steel surface, the conditions are right for a diffusion-controlled corrosion mechanism to act, in which the aggressive species from the atmosphere (O_2_, H_2_O and Cl^−^) pass through the rust layer to interact with the underlying steel (Figure 38a). This situation seems to occur in relatively low Cl^−^-containing atmospheres. The steel corrosion process consists of the following reactions:

Steel starts to corrode according to the anodic reaction:

Fe→Fe^2+^ + 2e^−^, (1)

where the cathodic process consists of reduction of oxygen dissolved in the aqueous adlayer:

O_2_ + 2H_2_O + 4e^−^→4OH^−^, (2)

The OH^−^ ions formed migrate towards the anodic zones forming Fe(OH)_2_ as the initial rust product:

Fe^2+^ + 2OH^−^→Fe(OH)_2_, (32)

Under this basic mechanism, the steel corrosion rate will be highly influenced by the concentration of ionisable substances in the aqueous adlayer, as in the case of chlorides present in marine atmospheres. This explains the notable increase in the steel corrosion rate at station Cabo Vilano-2 compared to the Ponte Do Porto background station (Table 4), as the Cl^−^ deposition rate rises from 3.6 mg Cl^−^/m^2^·d at Ponte Do Porto to 70 mg Cl^−^/m^2^·d at Cabo Vilano-2.

In contrast, the exposure of CS to severe marine atmospheres can lead in certain circumstances to the formation of thick rust layers. High times of wetness of the metallic surface and an atmosphere with a high Cl^−^ deposition rate lead to the formation of this type of rust. These thick rust layers tend to become detached from the steel substrate, leaving it uncovered and without protection and thus accelerating the metallic corrosion process. The formation of anomalous thick rust layers and the accompanying exfoliation phenomenon has also been observed in studies carried out by the authors and other researchers [42,129,153,161].

In studies by Chico et al. on CS in Cl^−^-rich atmospheres, the average Cl^−^ deposition rate needed to exceed a critical threshold of close to 300 mg Cl^−^/m^2^·d for exfoliation to take place. The annual steel corrosion at that atmospheric salinity was higher than 100 μm [129].

Exfoliated rust layers are composed of multiple rust strata, as can clearly be seen in the cross-section of Figure 23. The characteristics of the different rust sublayers within the rust multilayer are described in Figure 24: the outermost rust layer (OR) (rich in lepidocrocite and goethite), and a succession of alternating strata of fragile compact rust (CR) and loose interlayer rust (LIR) layers [12,129,153,162].

The CRs present high goethite and maghemite contents, low lepidocrocite contents and the practical absence of akaganeite. The mechanism that is proposed for the formation of CRs consists of two stages: (i) the formation of magnetite by electrochemical reduction of lepidocrocite and akaganeite phases (wet stage); and (ii) the solid-state transformation of magnetite into maghemite (dry stage). The LIR presents high goethite and akaganeite contents along with low lepidocrocite and spinel contents. It is proposed that the akaganeite and lepidocrocite phases will be electrochemically reduced to magnetite (maghemite at a later stage) and the formation of the CR layer takes place by consumption of the akaganeite and lepidocrocite phases leading to the complete disappearance of the interlayer rust stratum. An extremely dry period may cause the corrosion process to end without fully exhausting the interlayer rust stratum.

**Figure 40:**
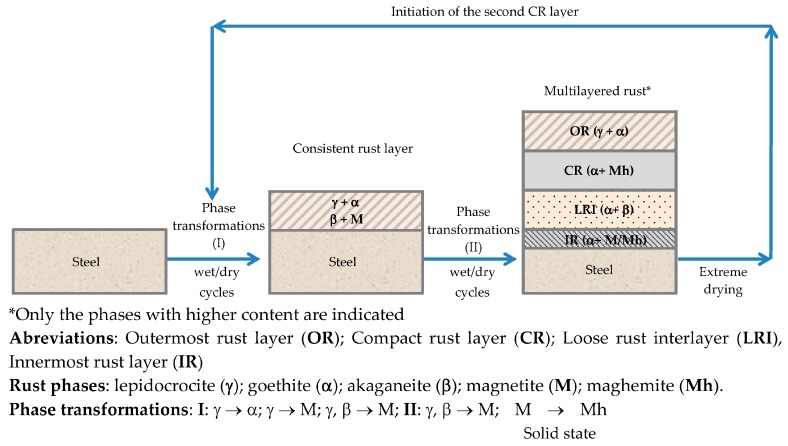
Scheme of a feasible multilayered rust formation mechanism of carbon steel exposed to severe marine atmospheres [129,153].

Subsequently, once the extremely dry period has come to an end and a new wet period starts, the formation of a second CR layer would begin, and so on, giving rise to the formation of a sandwich-type structure constituted by alternate CR and LIR layers. A scheme of a feasible multilayered rust formation and rust exfoliation mechanism for CS exposed to severe marine atmospheres is shown in Figure 40 [129,153].

The detachment (exfoliation) of multilayered rust from the steel substrate takes place after complete drying of the whole rust layer, creating expansion stresses that exceed the adhesion forces which keep the multilayered rust joined to the steel substrate.

The difference in density between the sublayers involved (CR and LIR) suggests that compactness combined with mechanical properties may play an important role in the triggering of rust exfoliation. A closer look at the molar volume of the rust phases, i.e., the ratio between the molar mass and the density of each phase expressed in cm^3^/mol, indeed shows great variations [162]. Table 5 displays a factor of 5 when going from the most compact rust phases involved (goethite, lepidocrocite; molar volume around 20 cm^3^/mol), over medium compact phases (magnetite, maghemite; around 40 cm^3^/mol), to the least compact phase (akaganeite; around 100 cm^3^/mol) [194]. The much lower compactness of akaganeite is due to the presence of tunnels of the akaganeite lattice into which the Cl^−^ ions can enter and become integrated, resulting in a much less dense structure than the other rust phases [162].

Taking this difference in molar volume into consideration, it is possible to anticipate a great volume contraction and consequent void formation when the least compact phase akaganeite is structurally transformed into the much more compact spinel phase during primarily wet periods. Similarly, great volume expansion and stress introduction is induced when lepidocrocite is transformed into spinel. Hence, considering the molar volume data, it is not surprising that the compact rust sublayer contains the rust phases with the lowest molar volume (goethite and spinel) while the loose rust interlayer is dominated by akaganeite with a higher molar volume than the main phases in the solid rust sublayer [162].

Thus it is suggested that rust exfoliation is the result of frequent phase transformations, together with great variations in compactness between the rust phases involved. At some critical point the changes in compactness, compressive stresses and void formation become too large and the whole rust sublayer collapses mechanically and results in a fracture along the innermost rust layer [162].

## 8. Coastal-Industrial Atmospheres

The atmosphere of many coastal cities in developing countries is polluted with SO_2_ due to the growth of industry, and in many cases formerly pure marine atmospheres can now be categorised as marine-industrial. The effect of SO_2_ on the corrosion behaviour of steel in atmospheres containing Cl^−^ has not been widely studied. The first information on this subject was published by Copson [195] in 1945, who reported that a combined influence of Cl^−^ deposited on the surface and SO_2_ in the atmosphere was considered to cause extensive corrosion. The first laboratory research was carried out by Ericsson [196], who observed a synergic effect of the combined influence of SO_2_ (1 mg SO_2_/cm^2^·h) and NaCl (8 mg NaCl/cm^2^) at 90% RH which was not seen at 70% RH.

The small amount of research that has been performed on this matter has been carried out in field tests by Corvo [80], Allam [197], Almeida et al. [198], Feliu and Morcillo [5], Liang et al. [199] and Wang et al. [200].

Corvo [80], after 6 months of atmospheric exposure of steel at marine testing stations in Cuba, found the following damage function:

C(g/m^2^) = 64.9 + 6.9 [Cl^−^] + 0.15 [SO_2_]^2^ − 0.17 [Cl^−^] [SO_2_]^2^, (33)

From which a very significant influence of Cl^−^ is deduced, according to the coefficient obtained. SO_2_ also influences weight loss with a lower coefficient, but in a quadratic form. However, the combined influence of SO_2_ and Cl^−^ has a negative sign, indicating a decrease in corrosion. The results corresponding to multilinear stepwise regression and correlation for annual data are however different, where coefficients affecting SO_2_ alone or combined with Cl^−^ are not significant.

C(g/m^2^) = 243.7 + 6.7 [Cl^−^], (34)

Allam [197], on the basis of results obtained in a study carried out at the shoreline on the western coast of the Arabian Gulf, with the presence of SO_2_ (10 ppb) and H_2_S (70 ppb) in the atmosphere, formulated the following mechanism for tests from 10 h to 12 months in duration, in which advanced surface analysis was used to characterise the corrosion products.

During the initial stage, the formation of iron sulfate (FeSO_4_) takes place concurrently with the formation of iron chlorides. The negatively charged sulfate ions may compete with Cl^−^ ions for the ferrous ions (Fe^2+^) produced by anodic reaction. This competitive effect has been reported in different papers [70,201,202].

Fe^2+^ + 2Cl^−^→FeCl_2_, (35)

Fe^2+^ + SO_4_^2−^→FeSO_4_, (36)

Iron chlorides, as a major constituent in the initially formed blister covers, indicate that Cl^−^ ions are more aggressive than sulfate ions during the initial stages of AC. During the formation of FeSO_4_ at anodic sites, sodium ions (Na^+^) in the electrolyte are expected to migrate to the cathodic sites at the periphery of the anodic sites (blister site) to form sodium sulfate (Na_2_SO_4_). As blisters grow to form a thick continuous corrosion product layer, the formation of iron chlorides will eventually decrease compared to that seen during the initial stages. In contrast, further formation of iron sulfates takes place at the metal/rust interface during prolonged exposure times.

Feliu and Morcillo [5] in mixed atmospheres with SO_2_ and NaCl contents ranging from 0.2–1.6 mg SO_2_/dm^2^·d and 0.5–3.8 mg NaCl/dm^2^·d, respectively, observe an additivity of effects as both pollutants act together.

**Figure 41:**
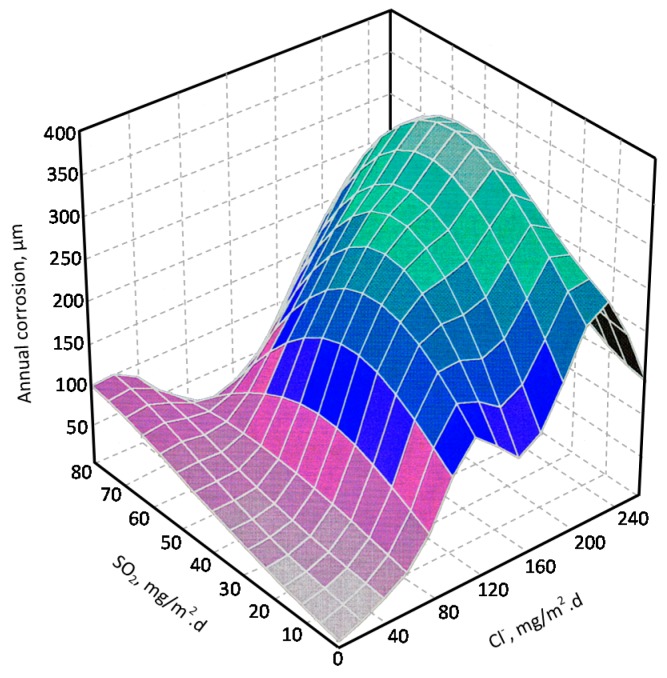
3D representation of annual steel corrosion rate as a function of SO_2_ and Cl^−^ contents in the atmosphere [198].

Almeida et al. [198] carried out a study involving a large number of atmospheres in the Ibero-American region in which both pollutants were found: Cl^−^ ranging from 4.4 to 203.0 mg Cl^−^/m^2^·d and SO_2_ ranging from 16.7 to 65.2 mg SO_2_/m^2^·d. Figure 41 shows the evolution of CS corrosion with the atmospheric concentration of both pollutants, in which it is possible to see the significantly more corrosive action of Cl^−^ compared to SO_2_. At low Cl^−^ contents the presence of SO_2_ shows a beneficial effect on the corrosion of the base steel, as SO_2_ favours the transformation of lepidocrocite into goethite. As the Cl^−^ concentration rises, the attack is intensifies with the SO_2_ content (perhaps a synergic effect) and among the corrosion products it is possible to see the presence of akaganeite together with the lepidocrocite and goethite phases. After passing a certain threshold in the concentration of both pollutants, the attack of the steel seems to decrease, but this observation will need to be confirmed in a greater number of atmospheres with very high concentrations of both pollutants.

Liang et al. [199] carried out a sixteen-year AC exposure study of steels and found that SO_2_ only had an obvious deteriorating effect in the initial stages of atmospheric exposure. Wang et al. [200] also confirm this effect, indicating that Cl^−^ is the ion that plays the main role in later stages, contrarily to the findings of Allam [197].

Some laboratory studies have also been carried out on the joint effect of both pollutants acting in combination. Attention is drawn to the contributions of Knotková et al. [203], Bastidas [204] and Chen et al. [205].

Knotková et al. [203] studied the joint action of both pollutants by alternate exposure of steel in cabinets containing SO_2_ and sprayed NaCl solution (100–200 mg NaCl/m^2^.d or equivalent Cl^−^ contents in artificial seawater), respectively. They also studied the effect of SO_2_ using Na_2_SO_4_ solutions. They saw that (a) the corrosion products formed exhibited sulfate nests and akaganeite, the latter in smaller quantities than when the Cl^−^ pollutant acted individually. From the morphological point of view, the influence of Cl^−^ was predominant; and (b) a synergic effect on steel corrosion which would disappear by the end of the test, and their effect would only be additive.

These researchers conclude that the steel corrosion process when the two pollutants act together is a complex process involving two fundamental factors: the activity of H^+^ in the aqueous adlayer formed on the metal

SO_2_ + H_2_O + ½O_2_→H_2_SO_4_, (37)

Which acquires an acid pH, and the ion capturing capacity of the corrosion products that are formed. In this respect they note that when SO_4_^2−^ is introduced in the form of sodium sulfate (Na_2_SO_4_) solution there is an increase in the pH value, an opposing effect to the situation noted above, as a consequence of the formation of a very stable NaOH solution:

Na_2_SO_4_→2Na^+^ + SO_4_^2−^, (38)

Fe + SO_4_^2−^→FeSO_4_ + 2e^−^, (39)

½O_2_ + H_2_O + 2e^−^→2OH^−^, (40)

**Figure 42:**
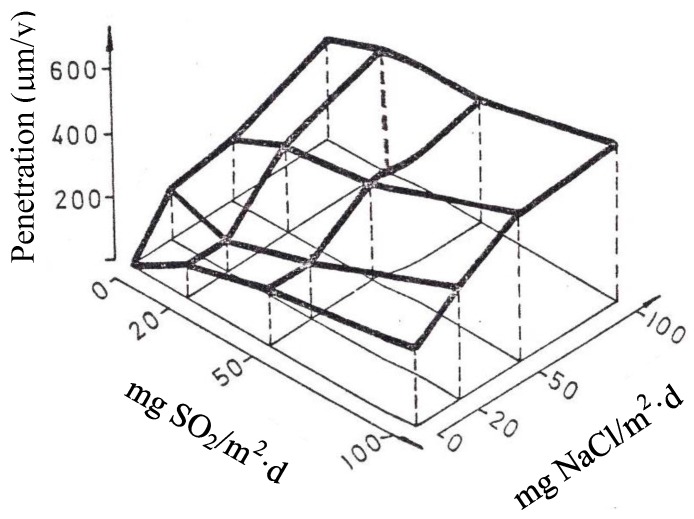
Effect of chlorides, SO_2_ and the combination of both pollutants on iron corrosion kinetics. Electrochemical testing in conditions of 100% RH and 30 °C temperature [204].

Bastidas [204], in alternate exposure to NaCl (20, 50 and 100 mg/m^2^·d) and iron sulfate at the same concentrations, observe that Cl^−^ is more harmful than an identical concentration of SO_2_, and that the presence of one pollutant increases the attack caused by the other; the higher the concentration, the greater the combined effect (Figure 42). They find an additivity of effects when both pollutants act together.

Finally, Chen et al. [205] have recently carried out a wet/dry cyclic corrosion test using electrolytes prepared by adding different amounts of Na_2_SO_3_ (from 25.6 to 256 mg/m^2^·d) into a NaCl solution (710 mg/m^2^·d). They note that in the co-presence of Cl^−^ and SO_2_, the steel corrosion mass gain increases as the SO_2_ content rises up to a certain level, and beyond this level the steel shows a slower mass gain. From the kinetic point of view this finding is not in accordance with the commonly held idea [30] that a higher SO_2_ content in the atmosphere should lead to a higher steel corrosion rate. Cl^−^ dominates the corrosion process in the initial stage and the effect of SO_2_ comes later, accelerating the corrosion process as Allam reported [197].

Thus, this is a topic of great practical importance where, as has been seen, there is still considerable controversy on the effect of SO_2_ in relation with the effect of Cl^−^ in the initial stages of the AC process and the total magnitude of the corrosive attack with exposure time. Accordingly, greater research efforts are needed on both aspects.

## 9. Long-Term Behaviour of Carbon Steel Exposed to Marine Atmospheres

For socio-economically advanced societies with heavy infrastructure investments in coastal regions, steel corrosion may be a considerable problem. Thus it is fundamental for engineers and political policy-makers to be able to predict AC well into the future (25, 50, 100 years). It must be considered that in some highly developed countries efforts are now being made to design civil structures such as bridges and other load-bearing structures for 50–100 years of service without any maintenance. Data mining and modelling tools can help to improve AC forecasts and anti-corrosive designs, but despite great progress in the development of damage functions (dose-response) in wide-scale international cooperative research programmes there is still a way to go for such long-term modelling of AC processes.

### 9.1. Nature of Corrosion Products

The nature of the rust constituents is barely affected by the exposure time; in fact, the same species are usually detected at a given site however long the exposure. The time factor only alters the proportions of the constituents, or at most determines the appearance or disappearance of intermediate or minor compounds [97]. Thus, lepidocrocite, goethite, akaganeite and spinel phase (magnetite/maghemite) are usually the main corrosion products found on steel after long-term marine atmospheric exposure. Akaganeite is a typical component of rust developed in marine atmospheres. On contacting the steel surface, akaganeite is gradually transformed into magnetite [121,122], in such a way that in severe marine atmospheres this substance can become the main component of the corrosion layer [124].

### 9.2. First Year Steel Corrosion

There are numerous published damage functions on CS corrosion and environmental parameters, both meteorological (air temperature, RH, rainfall, TOW, etc.) and atmospheric-pollution-related (mainly SO_2_ and airborne salinity). In this respect, attention is drawn to the efforts made by ISO with regard to atmospheric corrosivity classification (ISO 9223 [30]) and the international cooperative programmes on AC: ISOCORRAG [29], ICP Materials [206], and MICAT [207].

In Section 4 it was seen that the annual corrosion of steel accelerates as the saline content in the atmosphere rises (Figure 7); the magnitude of the attack in marine atmospheres normally exceeds that found in other types of atmospheres.

### 9.3. Long-Term Steel Corrosion

For long-term AC, most of the experimental data has been found to adhere to the following kinetic relationship:

C = A*t*^*n*^, (41)

where C is the corrosion after time *t*, and A and *n* are constants.

Thus, corrosion penetration data is usually fitted to a power function involving logarithmic transformation of the exposure time and corrosion penetration:

log C = log A + *n* log *t*, (42)

**Figure 43:**
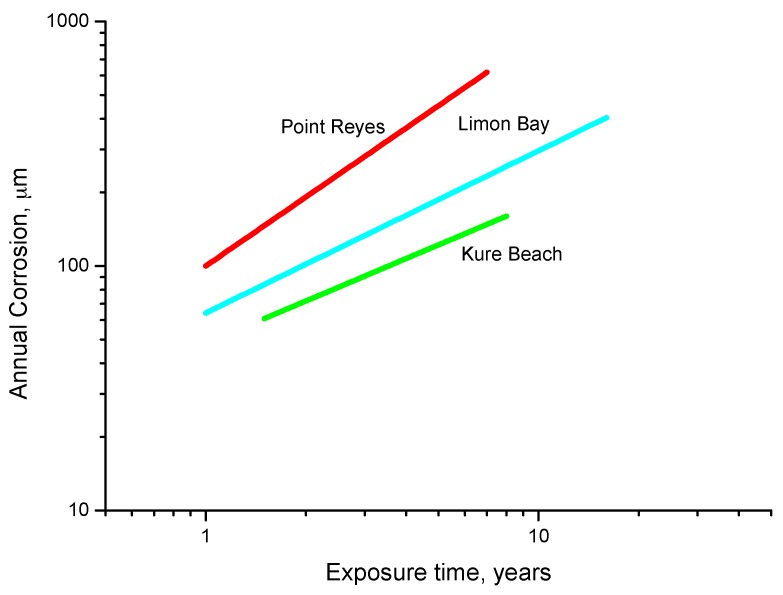
Typical log-log plots for carbon steel corrosion versus exposure time at different marine sites: Kure Beach (USA), Limon Bay (Panamá) and Point Reyes (USA). Data obtained from the reference [87].

This power function (also called the bilograrithmic law) is widely used to predict the AC behaviour of metallic materials even after long exposure times, and its accuracy and reliability have been demonstrated by a great number of authors. Figure 43, obtained from CS corrosion data after different exposure times in marine atmospheres at different test sites [87], confirms the verification of the power function (Equation (41)).

According to Benarie and Lipfert [208], Equation (41) is a mass-balance equation, showing that the diffusion process is rate-determining, and this rate depends on the diffusive properties of the layer separating the reactants. The exponential law with *n* close to 0.5, can result from an ideal diffusion-controlled mechanism when all the corrosion products remain on the metal surface. This situation seems to occur in slightly polluted inland atmospheres. On the other hand, *n* values of more than 0.5 arise due to acceleration of the diffusion process (e.g., as a result of rust detachment by erosion, dissolution, flaking, cracking, etc.). This situation is typical of marine atmospheres, even those with low Cl^−^ contents. Conversely, *n* values of less than 0.5 result from a decrease in the diffusion coefficient with time through recrystallisation, agglomeration, compaction, etc. of the rust layer. Therefore, the exponent *n* value can be used as an indicator of the physico-chemical behaviour of the corrosion product layer and thus of its interaction with the local atmosphere, exposure conditions, nature of wetting/drying cycles, etc.

According to Benarie and Lipfert [208], as a rule *n* < 1 and there is no physical sense for *n* > 1, as *n* = 1 is the limit for unimpeded diffusion (high permeable corrosion products or no layer at all). Thus, values of *n* > 1, very frequent in severe marine atmospheres, have been dismissed in many MAC studies as being due to outliers or errors in mass loss determinations.

**Figure 44:**
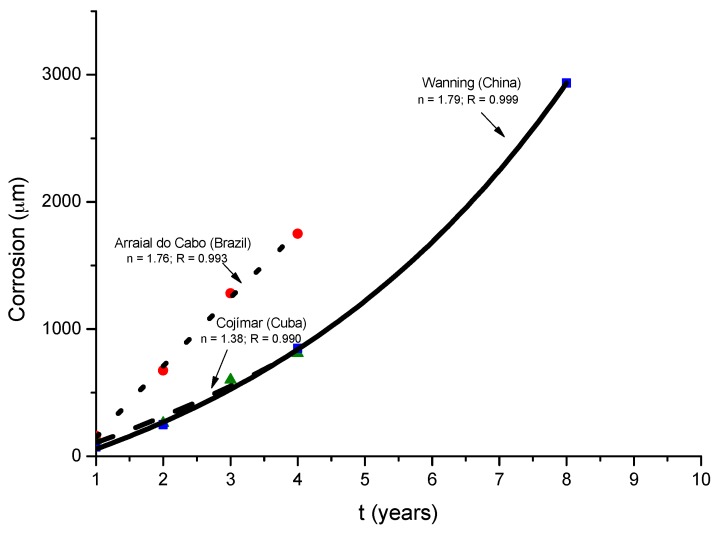
Evolution of mild steel corrosion with exposure time in severe marine atmospheres: Wanning, China, Arraial do Cabo, Brazil and Cojímar, Cuba. Values of exponent n and correlation coefficient (R) have been obtained from log C vs. log t plots for each site [12].

However, many of these may be real values and not be due to error in mass loss determinations. The reason for this behaviour lies in the fact that in highly severe marine atmospheres, Equation (41), based on diffusion mechanisms, can sometimes not be applicable. When applied, it is common to find exponent *n* values of close to 1 due to the existence of highly permeable (and barely protective) corrosion layers or the absence of corrosion layers because of their detachment by delamination (exfoliation), or even values of *n* > 1 due to acceleration of the corrosive attack as a result of an “autocatalytic” mechanism [23], in contrast to the diffusion mechanism upon which Equation (41) is based. Figure 44 shows the evolution of steel corrosion with exposure time in marine atmospheres with high deposited Cl^−^ ion contents. The acceleration of the attack as exposure time advances is evident [12].

**Figure 45:**
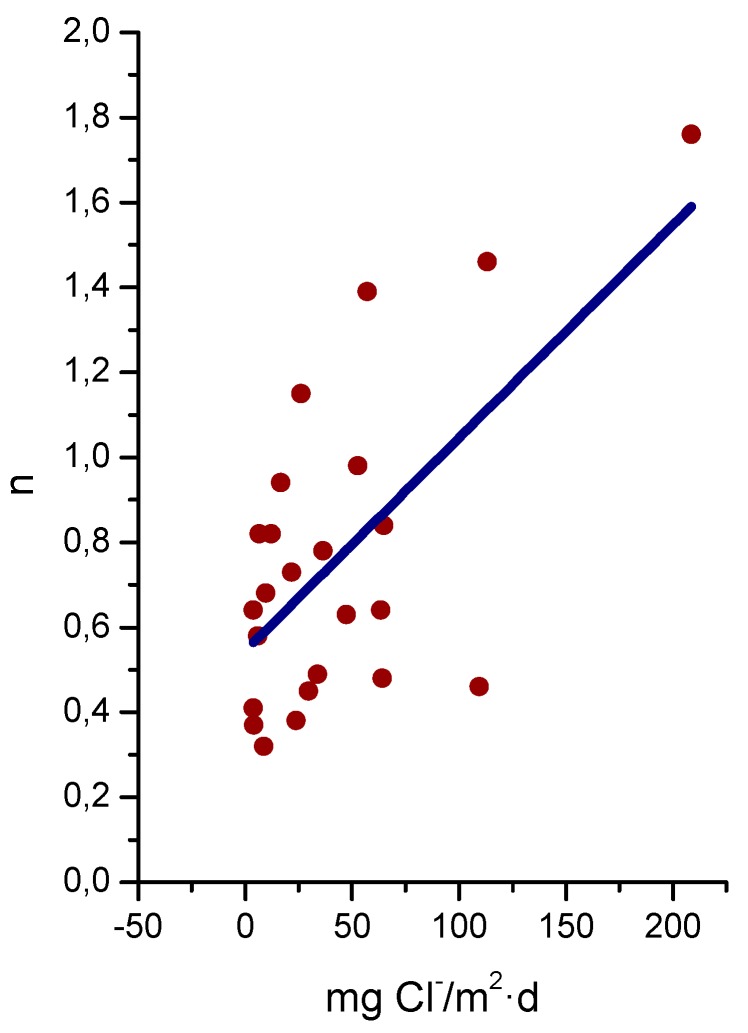
Variation in exponent n with atmospheric salinity at marine test sites in the MICAT [207] and ISOCORRAG [29] programmes.

In an attempt to relate the exponent *n* value with atmospheric salinity, Figure 45, prepared using data obtained in the ISOCORRAG [29] and MICAT [207] programmes, for marine atmospheres with low SO_2_ levels (<35 mg SO_2_/m^2^·d), shows the tendency for the exponent *n* value to increase with atmospheric salinity and how *n* can acquire values of more than unity. It would be important to have a greater volume of data for *n* > 1 in order to perfect Figure 45 [12].

Highly permeable corrosion layers or the absence of thick corrosion layers at all because of their detachment by delamination (exfoliation) (see Figure 38) must be constituted by macropores (2 < D < 10 nm) or perhaps even mesopores (D ~ 10–30 nm), instead of micropores (D < 2 nm), as occurs in consolidated rust layers formed in marine atmospheres of less aggressivity. According to Ishikawa et al. [146], macropores are considered to be too large to govern diffusion of molecules and ions through the rust layers.

### 9.4. Modelling of the Long-Term Atmospheric Corrosion Process

Data on the corrosion resistance of metals over long periods of time is important for determining the service life of metal structures and for developing the methods and means for their protection and preservation. Reliable estimates of corrosion resistance can be provided by corrosion tests under natural conditions. Such tests are time-consuming and expensive. In view of this, researchers pay great attention to the development of models that allow long-term forecasts without requiring testing under natural conditions.

**Figure 46:**
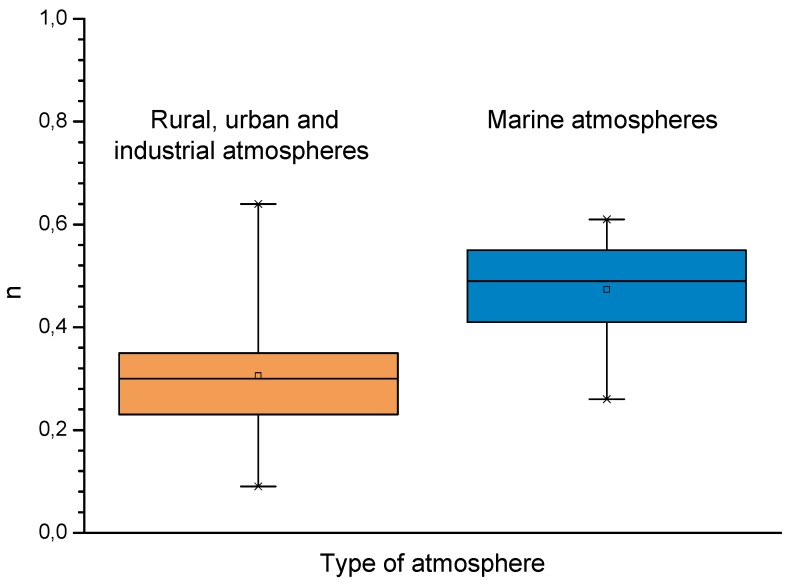
Box-whisker plots of n values in power function (C = At^n^) for carbon steel in different types of atmospheres [87].

It has been seen that the power function (Equation (41)) is widely used in long-term forecasts of the AC of metals. Table 6 sets out average values of exponent *n* for plain CS in different types of atmospheres, and Figure 46 shows the corresponding box-whisker plots of *n* values. It is possible to see a clear tendency towards higher *n* values in marine atmospheres.

Panchenko et al. [209] propose a modification of the model for the long-term forecasting of corrosion losses of metals in any type of atmosphere after the establishment of the steady state. They find a stochastic relationship between exponent *n* of the power function (Equation (41)) and corrosion losses over the first year, and make a forecast of corrosion losses based on a power function using the *n* values calculated from the identified stochastic relationships.

McCuen and Albrecht [210] proposed improving the power model by replacing it with two different approaches: numerical model and power-linear model, the latter consisting of a power function at the initial stage (Equation (41)) and a linear function:

C_t_ = C_0_ + α_t_, (43)

At the steady-state stage.

As to whether this law provides a better prediction of the AC of WS for exposure times of at least 20 years, McCuen et al. compared both models (the power model and the power-linear model) using AC data reported for WS in the United States and concluded that the experimental data fitted the power-linear model better than the power model and thus provided more accurate predictions of long-term AC [210].

Panchenko et al. [211] propose methods for the calculation of *n* and *α* (Equation (43)) and make a comparative estimate of long-term predictions using the power-linear function and ISOCORRAG standard, obtaining comparable results in atmospheric corrosivity categories C1–C3 [30].

Albrecht and Hall [212], by refinement of the power-linear model, have proposed a new bi-linear model based on ISO 9224 [213], called modified ISO 9224, as well as an adjustment of this new bi-linear model that accounts for a modified corrosion rate during the first year of exposure and a steady state in subsequent years.

**Figure 47:**
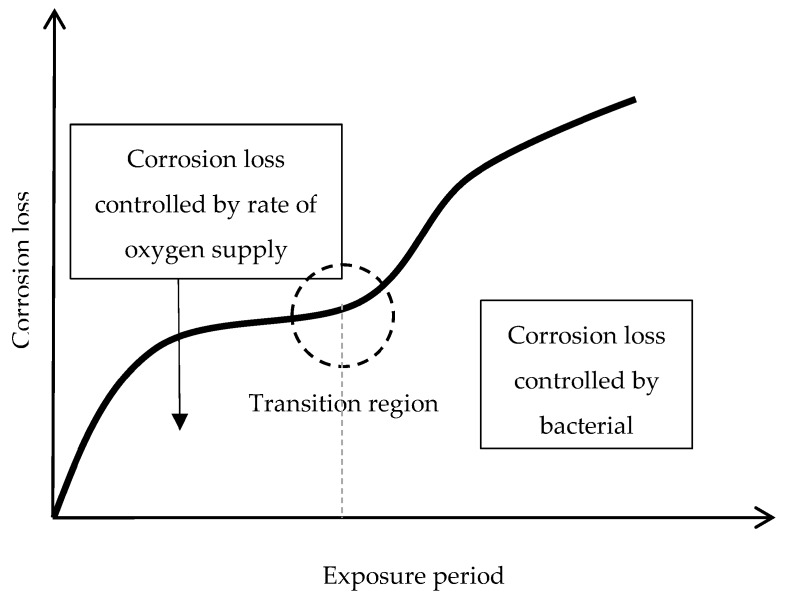
Bi-modal model for corrosion loss showing the changing behavior of corrosion process, according to Melchers [214,215].

Finally, Melchers [214,215] suggests a bi-modal model for long-term forecasts of the corrosion loss of WS and grey cast iron in marine atmospheres. The model consists of a number of sequential corrosion phases, each representing the corrosion process that is dominant at that time and which controls the instantaneous corrosion rate. The phases are summarised in Figure 47. The important difference from conventional models is that the bi-modal model has longer-term corrosion governed by microbial activity. Melchers has successfully applied this model to different sets of data points for long-term exposures at different test sites.

### 9.5. Towards an Estimation of the Marine Atmospheric Corrosion of Steel Based on Existing Environmental Parameters

Following the philosophy of ancient ISO 9223 [216], an estimation of the atmospheric corrosivity of a particular coastal region could be made from the knowledge of three fundamental parameters: TOW of the metallic surface, SO_2_ concentration in the atmosphere, and Cl^−^ deposition rate due to airborne sea-salt particles.

Nowadays most countries have ample meteorological databases covering their entire territories which would allow estimations of TOW. Furthermore, information on atmospheric SO_2_ concentrations is increasingly available. However, there tends to be very little information available on atmospheric salinity in different coastal areas, and reliable databases on this subject do not generally exist. It would be desirable to include this data in the numerous published damage functions between steel corrosion and environmental factors. This would make it possible to estimate AC simply from environmental data, without having to carry out natural corrosion tests at a specific site, which involve long waiting times and considerable expense.

#### 9.5.1. Time of Wetness

TOW can generally be defined as the amount of time a metal surface remains wet during atmospheric exposure. The most commonly used practical definition of TOW is that given by ISO 9223 [30], which defines it as the total time when the RH of the ambient environment is equal to or greater than 80% at temperatures above 0 °C.

A number of limitations of the ISO definition have been pointed out in the literature and even in the ISO 9223 standard itself: (a) One major issue is that by definition precipitation and dewing events are excluded in ISO 9223. With regard to the latter, temperature differences between the ambient air and the surface can cause quite large deviations between the RH of the surface and the surrounding air; and (b) many atmospheric pollutants deliquesce at humidity levels far below 80%. A study by Cole et al. [13] clearly shows wetness measured on resistance-type sensors occurring well below the 80% threshold. Wetting phenomena associated with AC along with TOW definitions and determination methods were overviewed by Schindelholz and Kelly [14,15,16].

According to Cole et al. [13], in the case of salt deposits on the metallic surface, as occurs in marine atmospheres, it is necessary to take into account the simple principle that a metal surface is wetted when the surface RH exceeds the deliquescent RH (DRH) of any salts on the surface, as the ambient RH increasingly exceeds the DRH. They note the need for a more flexible method for predicting not only TOW but also the cycles of moisture accumulation and depletion. Given the established role of hygroscopic salts in promoting wetting in particulates, whether airborne or on surfaces, a model of surface wetting should address the role of deposited salts. The basic principle of the model is that the TOW of metal surfaces fully exposed to the environment can be approximated by the time of condensation (TCD) plus rain periods, i.e.,

TOW = TCD + rain period,(44)

Thus, these researchers propose a method for estimating the wetting of a surface based on a comparison of surface RH and the deliquescence of salts that may pollute the surface, deriving relatively simple rules for wetting based on the DRH model and ISO classifications. These rules predict the total TOW to a high degree of accuracy.

#### 9.5.2. Chloride Deposition Rate

Studies have shown that the two main sources of salt aerosol carried by the wind are ocean waves and breaking surf [60,66,217,218,219]. The magnitude of atmospheric salinity (Cl^−^ deposition rate) at any given place depends on numerous factors. To mention just a few (see Section 4): wave height at high sea and at the coast; presence or absence of a surf zone on the coast; direction, speed and persistence of marine winds; height above sea level; distance from the shore; topographical effects; etc. Thus it has been seen in Section 4 that for instance wave height values of 1.5–2.0 m are sufficient to produce high monthly average salinity values, or that the wind blowing for a relatively short time at speeds above 3 m/s in a direction with great influence on the entrainment of marine aerosol is sufficient for atmospheric salinity to acquire important values.

There is abundant literature on this topic. Perhaps a less studied aspect has been the degree of shielding or sheltering on wind speed. In relation with this matter, Nakajima [220] has developed a mapping method based on grids with topographic factors to assess the influence of several factors on average wind speed at locations near a sea coast. The concept integrates the geographical texture of the environment in all directions with its effect on airflow. The most significant factor affecting sea wind is the degree of shielding.

Similarly, Klassen and Roberge [221] have measured and modelled by CLIMAT units [222] the influence of wind effects on local atmospheric corrosivity considering various degrees of wind sheltering. They found a 34-fold difference between the average mass loss of the most wind-protected and the least wind-protected points.

As noted by Roberge et al. [223], the dispersion of airborne salinity is highly dependent upon the local geography and wind patterns and is therefore rather difficult to model with simplistic functions. In this respect, Cole et al. [218] developed an aerosol penetration model applying the principles of aerosol transport using fluid dynamics principles. Salt aerosol sinks, such as gravity, rain and trees (obstacles), were accounted for. The study also noted that a high surface RH and a lower cloud height led to decreased penetration inland, and further reductions occurred in areas of high rainfall. It was also noted that structures in the airflow, such as forests and urban environments, reduced the Cl^−^ concentration. This work illustrated that complex variables such as airspeed, ground roughness (vegetation), surface air RH, cloud height and rainfall could be incorporated into a model. A good correlation was reported to exist between the model and empirical results from a limited data set.

Despite the high number of factors involved, it is hoped that the growing flow of knowledge on this subject will soon lead to the desirable goal of being able to make a rough estimate of atmospheric salinity in a given geographic area without the need for measurements involving marine aerosol capturing techniques.

The prediction of atmospheric corrosivity at a given site is even more complex, as it is necessary to take into account an even greater number of variables. Thus, Roberge et al. paint a pessimistic view when they note that a single transferable and comprehensive environmental corrosivity prediction model still has to be published and may not be possible due to the complexity of the issues [223].

## 10. Issues Pending

AC has been extensively researched over the last one hundred years, and as a result the effects of meteorological and pollution variables on AC are now well known. Even so, our knowledge on this issue still holds many gaps, such as how to accurately estimate the total TOW of metallic structures, and the effects of climate change and acid rain, etc.

The issue of steel corrosion in coastal regions is particularly relevant in view of the latter’s great importance to human society, considering that about half of the world’s population lives in coastal regions. Thus it is surprising that marine atmospheric corrosion (MAC) has until recently received relatively little attention by corrosion scientists. This is therefore a relatively young scientific field, where there continue to be great gaps in knowledge.

In this review, we have noted a number of aspects in relation with which greater research efforts would seem to be necessary. To mention just a few:

1. Experimentation in this field is carried out at atmospheric corrosion testing stations and in the laboratory by means of wet/dry cyclic tests. A great amount of research is under way using both methods, but there are two issues of enormous importance that are not being paid sufficient attention: (a) experimentation in marine atmospheres with high Cl^−^ ion deposition rates (>500 mg/m^2^·d), where very little information is available; and (b) the standardisation of wet/dry laboratory cyclic tests by means of more specific codes in order to make research results more comparable.
  It would also be necessary to undertake more research in the case of marine-industrial atmospheres, where there are great discrepancies among researchers. This is a particularly important issue in developing countries where factories are often located in coastal regions.
2. One currently unresolved question is concerned with the presence of the amorphous phase in the rust layer and the evolution of its amount with exposure time. With regard to the role played in corrosion mechanisms by the less crystalline phases of rust (ferrihydrite, feroxyhyte, etc.), despite the enormous research effort that has been carried out in recent years by a number of research teams using highly sophisticated analytical techniques, there are still numerous gaps in knowledge.
  Another matter that generates a great deal of uncertainty is the differentiation of magnetite and maghemite phases, both of which are very similar in many of their characteristics, but which can play a different role in the MAC process.
3. In the rust layers formed, aspects such as decoupling of the anodic and cathodic corrosion reactions, localisation, connectivity and reactivity of the different rust phases inside the corrosion layers, as well as characteristics such as porosity, tortuosity, etc., are also of primary importance in the determination of corrosion mechanisms.
  At high Cl^−^ ion deposition rates, rust layers can exfoliate and become detached from the steel substrate. Although great advances have recently been made in this field, there are still a number of basic aspects that remain to be clarified in order for a complete comprehension of rust exfoliation phenomena.
4. Finally, a matter of enormous technical importance for engineers and political policy-makers is to be able to predict steel corrosion rate well into the future (20, 50, 100 years). Data mining and modelling tools can help to improve forecasts and anti-corrosive designs, but despite great progress in the development of damage functions (dose-response) in wide-scale international cooperative research programmes, there is still a long way to go for such long-term modelling of atmospheric corrosion processes.
  In this sense, better scientific knowledge is needed towards the desirable goal of being able to estimate atmospheric salinity in a given geographic area without the need for measurements involving marine aerosol capturing techniques.

## Figures and Tables

**Table 1:** Iron corrosion species according Cornell and Schwertmann [91].

Type	Name	Formula
Oxides	Magnetite	Fe_3_O_4_
	Maghemite	γ-Fe_2_0_3_
	Hematite	α-Fe_2_0_3_
Hydroxides	-	Fe(OH)_2_
	Bernalite	Fe(OH)_3_
	Green rusts	Fe_x_^III^ Fe_y_^II^ (OH)_3x+2y−z_ (A^−^)_z_ where A^−^ = Cl^−^; ½SO_4_^2−^
	Ferrydrite	Fe_5_O_8_H·H_2_O
Oxyhydroxides	Goethite	α-FeOOH
	Lepidocrocite	γ-FeOOH
	Akaganeite	β-FeOOH
	Feroxyhite	δ-FeOOH
	Schwertmannite	Fe_16_O_16_(OH)_y_(SO_4_)_z_·nH_2_O

**Table 2:** Iron corrosion species containing chloride [92].

Name	Formula
Ferrous chloride (lawrencite)	FeCl_2_
Ferric chloride (molysite)	FeCl_3_
Ferric oxychloride	FeOCl
Ferrous hydroxychloride	β-Fe_2_(OH)_3_Cl
Green rusts	GR1 (GR Cl)
β-oxihydroxide (akaganeite)	β-FeOOH

**Table 3:** Principal habits (morphologies) of iron oxides according to Cornell and Schwertmann [91].

Iron Oxide	Morphology
Lepidocrocite (γ-FeOOH)	Laths
Goethite (α-FeOOH)	Acicular
Akaganeite (β-FeOOH)	Rods, somatoids
Feroxyhyte (δ-FeOOH)	Plates
Magnetite (Fe_3_O_4_)	Octohedra
Maghemite (γ-Fe_2_O_3_)	Laths or cubes
Hematite (α-Fe_2_O_3_)	Hexagonal plates, rhombohedra
Ferrihydrite (Fe_5_HO_8_·4H_2_O)	Spheres

**Table 4:** Variation of rust phases content on mild steel exposed during one year in test sites with different chloride deposition rate [64,150,193].

Test Site	Annual Average Chloride Deposition Rate, mg/m^2^·d	wt %			
		Lepidocrocite	Goethite	Akaganeite	Spinel
Ponte do Porto	4	100	*	0	0
Cabo Vilano-1	30	80.0	16.0	0	4.0
Cabo Vilano-2	70	59.6	20.0	17.6	2.7
Cabo Vilano-3	665	35.8	27.0	12.5	24.7

* The authors not ruling out a small contribution of this phase.

**Table 5:** Molar volume of different rust phases. Data from Crystallographic and Crystallochemical Database for Minerals and their Structural Analogues, WWW-MINCRYST. Institute of Experimental Mineralogy, Russian Academy of Sciences [194].

Phase	Molar Volume (cm^3^/mol)
Goethite (α-FeOOH)	20.84
Lepidocrocite (γ-FeOOH)	22.40
Maghemite (γ-Fe_2_O_3_)	43.71
Magnetite (Fe_3_O_4_)	44.56
Akaganeite (β-FeOOH)	101.62

**Table 6:** Average values of exponent n in bi-logarithmic plots of the power function (C = At^n^) for plain carbon steel in non-marine (rural-urban-industrial) and marine atmospheres [87].

Non-Marine (Rural-Urban-Industrial) Atmospheres		Marine Atmospheres	
*Av. n*	Range of *n* in Equation (41)	*Av. n*	Range of *n* in Equation (41)
0.49	0.26–0.76	0.73	0.37–0.98
